# The CST complex facilitates cell survival under oxidative genotoxic stress

**DOI:** 10.1371/journal.pone.0289304

**Published:** 2023-08-17

**Authors:** Tomohiko Hara, Hidenori Nakaoka, Tomoicihiro Miyoshi, Fuyuki Ishikawa

**Affiliations:** 1 Department of Gene Mechanisms, Graduate School of Biostudies, Kyoto University, Kyoto, Japan; 2 Radiation Biology Center, Graduate School of Biostudies, Kyoto University, Kyoto, Japan; 3 Laboratory for Retrotransposon Dynamics, RIKEN Center for Integrative Medical Sciences, Yokohama, Japan; University of North Carolina at Charlotte, UNITED STATES

## Abstract

Genomic DNA is constantly exposed to a variety of genotoxic stresses, and it is crucial for organisms to be equipped with mechanisms for repairing the damaged genome. Previously, it was demonstrated that the mammalian CST (CTC1-STN1-TEN1) complex, which was originally identified as a single-stranded DNA-binding trimeric protein complex essential for telomere maintenance, is required for survival in response to hydroxyurea (HU), which induces DNA replication fork stalling. It is still unclear, however, how the CST complex is involved in the repair of diverse types of DNA damage induced by oxidizing agents such as H_2_O_2_. *STN1* knockdown (KD) sensitized HeLa cells to high doses of H_2_O_2_. While H_2_O_2_ induced DNA strand breaks throughout the cell cycle, *STN1* KD cells were as resistant as control cells to H_2_O_2_ treatment when challenged in the G1 phase of the cell cycle, but they were sensitive when exposed to H_2_O_2_ in S/G2/M phase. *STN1* KD cells showed a failure of DNA synthesis and RAD51 foci formation upon H_2_O_2_ treatment. Chemical inhibition of RAD51 in sh*STN1* cells did not exacerbate the sensitivity to H_2_O_2_, implying that the CST complex and RAD51 act in the same pathway. Collectively, our results suggest that the CST complex is required for maintaining genomic stability in response to oxidative DNA damage, possibly through RAD51-dependent DNA repair/protection mechanisms.

## Introduction

The CST (CTC1-STN1-TEN1) complex is a hetero-trimeric, single-stranded DNA (ssDNA) binding protein complex. The first reports on the CST complex date back to a series of *in vitro* studies conducted in 1990, which showed that the complex (then called AAF for Alpha Accessory Factor) stimulates DNA polymerase α (pol α)/primase activities on ssDNA templates [1, 2]. Twenty years later, it was “rediscovered” as a telomere maintenance factor [3, 4]. Since then, its roles in the replication, protection, and length regulation of telomeres have been extensively studied [5–7]. These biological functions of CST at telomeres are thought to be mediated by two molecular functions: 1) ssDNA binding by oligonucleotide/oligosaccharide-binding (OB) fold domains [8]; and 2) recruitment and/or stimulation of DNA pol α/primase using templates of exposed ssDNA [4, 5, 9, 10]. Each of the three CST subunits have OB folds, which are structurally similar to those found in RPA (Replication protein A), a trimeric ssDNA-binding protein complex that plays a pivotal role in DNA metabolism, including replication, repair, and recombination [11, 12]. Whereas RPA is well known to exhibit little sequence preference, *in vitro* studies produced conflicting results concerning a proposed sequence preference of the CST complex: several reports showed a modest preference for GC-rich sequences while others did not [3, 5, 13–15]. Lagging strand synthesis by DNA pol α/primase is necessary to avoid telomere attrition caused by the “end replication problem”. The physical interaction between CST and DNA pol α is believed to be the basis of the efficient replication at the very ends of the chromosomes [16–18].

Our early work, involving immunofluorescence (IF) experiments in human cells, demonstrated that not all STN1 foci colocalized with telomeres, hinting at non-telomeric functions of CST [3]. Indeed, a growing amount of evidence now suggests that CST contributes to the integrity of the entire genome, and its role is not limited to telomere maintenance. Depletion of the CST complex in human cells did not affect cell cycle progression in normal conditions, but inhibited replication fork restart after hydroxyurea (HU) treatment [19]. HU depletes the dNTP pool, thereby inhibiting DNA replication fork progression. DNA fiber assays revealed that after the HU treatment, sh*STN1* cells exhibited a reduced frequency of late/dormant origin firing [19]. It is known that late/dormant origins contribute to rescuing stalled replication forks [20, 21]. It was proposed that the CST complex contributes to late/dormant origin firing by recruiting and/or stimulating DNA pol α/primase at the origins [22]. In a ChIP-seq study, CST was found to be localized to repetitive and GC-rich sequences throughout the genome in response to HU treatment [23]. CST-targeted loci were frequently colocalized with abnormalities (e.g. breakage, loss, and aberrant sister chromatid cohesion) in FISH (Fluorescence in situ hybridization) signals, suggesting that CST binds to fragile sites in the genome to prevent and/or repair DNA damage induced by compromised replication. Indeed, it was reported that rDNA arrays were unstable in the absence of the orthologous CST complex in fission yeast [24]. Replication fork stalling can lead to formation of ssDNA due to the halting of lagging strand synthesis, and RPA and RAD51 bind to such exposed ssDNA [reviewed in 25]. RAD51 is a key protein in homologous recombination (HR), where it forms nucleoprotein filaments along the exposed ssDNA prior to strand invasion of double-stranded template DNA [reviewed in 26]. In the context of replication fork stalling, RAD51 promotes fork reversal and protects the reversed fork structures, facilitating strand invasion for fork restart [20, 27]. Several reports demonstrated physical interactions between CST and RAD51, suggesting that CST recruits RAD51 to the stalled replication sites [23, 28]. The interaction was shown to be ATR dependent; thus, the CST response to replication stress is likely controlled by the DNA damage/replication checkpoint [23]. Another recent *in vitro* study demonstrated that CST binds specifically to unique ss/dsDNA structures that resemble a reversed replication fork, thereby protecting it from degradation by MRE11 nuclease [29]. The study showed that the amount of MRE11 bound to stalled forks increased in CST deficient cells, again consistent with the notion that an important *in vivo* function of CST is to stabilize stalled forks. Taken together, an emerging picture is that the CST complex preserves genomic integrity when cells are exposed to replication stress.

While the roles of the CST complex in the recovery from replication stress induced by HU have been extensively studied, the potential involvement of CST in the response to other genotoxic reagents still remains under investigated. In this work, we investigated phenotypes of sh*STN1* cells exposed to a high dose of hydrogen peroxide (H_2_O_2_), which potentially induces SSBs (DNA single-strand breaks) and DSBs (DNA double-strand breaks). We confirmed that 500 μM of H_2_O_2_ induced DSBs independently of DNA replication using the neutral comet assay and IF detection of γ-H2AX, and demonstrated that sh*STN1* cells were sensitive to that dose of H_2_O_2_. Cell synchronization experiments revealed that cells in S phase were particularly vulnerable to H_2_O_2_ while those at the G1/S boundary were insensitive to H_2_O_2_ even in the absence of STN1. We further showed that RAD51 accumulation was inhibited in the sh*STN1* cells treated with H_2_O_2_. These results suggest that the CST complex is required for RAD51-dependent DNA repair/protection particularly in S phase.

## Materials and methods

### Cell lines, culture conditions, and reagents

HeLa 1.2.11, HEK293T and U2OS cell lines were maintained at 5% CO_2_ and 37°C in Dulbecco’s Modified Eagle Medium (DMEM) (Nissui, Tokyo, Japan) supplemented with 10% fetal bovine serum (FBS) (Capricorn Scientific, Ebsdorfergrund, Germany), 1.92 mM L-glutamine (Sigma-Aldrich, St. Louis, MO, United States), 0.144% NaHCO_3_, 100 U/mL penicillin (Sigma-Aldrich) and 100 μg/mL streptomycin (Sigma-Aldrich). The VenorGeM Classic Mycoplasma Detection Kit (Sigma-Aldrich) was used to confirm that the cells were free of mycoplasma.

Hydrogen peroxide (H_2_O_2_) (FUJIFILM Wako Pure Chemical, Osaka, Japan) diluted in water, hydroxyurea (HU) (Sigma-Aldrich) diluted in water, Etoposide (Sigma Aldrich) and RAD51 inhibitor B02 (Cayman Chemical Company, Ann Arbor, MI, United States) in DMSO (Nacalai, Kyoto, Japan), and Mimosine (Cayman Chemical Company) in Phosphate-Buffered Saline (PBS) (Nissui) were added to culture media at concentrations indicated in each figure legend.

### Gene knockdown by shRNA

To construct shRNA-expressing plasmids for gene knockdowns, an annealed double-stranded synthesized DNA fragment containing the shRNA targeting sequence was inserted into the NotI and AgeI sites of a digested pLKO.1 vector (Sigma-Aldrich) using DNA Ligation Kit Mighty Mix (TaKaRa Bio, Shiga, Japan) at 15°C for 3–4 hours. *E*. *coli* XL1-Blue cells were transformed with the ligation products and plated on Luria Broth (LB) agar plates containing 50 μg/mL ampicillin. The inserted sequences were confirmed by sequencing from the U6 promoter sites. The parental pLKO.1 plasmid was utilized for the construction of a control cell line via lentiviral infection. shRNA sequences used are as follows: *CTC1* 5’-CAGGGAAATGACGACAATGAT-3’ [19]; *STN1* 5’-CAGCTTAACCTCACAACTTAA-3’; *STN1*#2 5’-CACTGGAGTTATAAACTGCAT-3’ [6, 23, 30]. Plasmids for transfection were purified using a GenElute HP Plasmid Midiprep Kit (Sigma-Aldrich).

To package lentiviral constructs, HEK293T cells were seeded in a 6-well plate at 2 x 10^5^ cells per well, with 2 mL of DMEM per well. Two days later, the cells were co-transfected with 4 μg plasmid DNA (2 μg of shRNA vector, 1 μg of pCAG-HIV gp, and 1 μg of pCMV-VSV-G-RSV-Rev) using 8 μg of 1 mg/mL transfection grade linear polyethylenimine hydrochloride (MW 40,000) (PEI-MAX-40K) (Polysciences, Warrington, PA, United States) in 1 mL of Opti-MEM. After overnight incubation, the culture medium was replaced with 3 mL of fresh DMEM. Approximately 48 hours post-transfection, the culture supernatant containing viral particles was harvested and filtered through a 0.45 μm polyethersulfone (PES) filter (Merck Millipore, Billerica, MA, United States). The supernatant was stored at -80°C until use.

To construct the constitutive knockdown cell lines for each assay, HeLa or U2OS cells were plated in a 6-well plate at 2 x 10^5^ cells per well. Two days later, the medium was replaced with medium containing the viral particles diluted 6-fold in fresh DMEM with 8 μg/mL polybrene (Sigma-Aldrich). After a 24-hour incubation, the culture medium was replaced with fresh DMEM containing 1 μg/mL puromycin (Sigma-Aldrich). After two days of drug-selection, cells were passaged and incubated an additional 2 days before the start of each assay.

### RNA extraction, reverse transcription, and qPCR

The shRNA expressing HeLa cells were plated in a 6-well plate at 2 x 10^5^ cells per well. After 2 days, the cells were washed with 1x PBS, and then 0.9 mL of TRIzol reagent (Thermo Fisher Scientific, Waltham, MA, United States) was added directly to each well. RNA extraction was performed according to the manufacturer’s instructions. One microgram of total RNA was used as a template in a reverse transcription reaction to synthesize cDNA, using an RNA PCR kit (TaKaRa Bio) according to the manufacturer’s protocol. The synthesized cDNA was quantified by quantitative PCR (qPCR) using a StepOne- Plus^™^ Real-Time PCR System (Thermo Fisher Scientific) and Luna Universal qPCR Master Mix (NEW ENGLAND BioLabs, Ipswich MA, United States). Primers used for qPCR are as follows: *CTC1* (forward 5’-TGGCTCTTCAGTCCGCTGGTTT-3’, reverse 5’-AACTCCAGAGGACGCCG-3’); *STN1* (forward 5’-GAGATTCATGCCACCGCTTAC-3’, reverse 5’-GCGCCTGGATTGCTTAGTG-3’) [19]; *GAPDH* (forward 5’-CCTGCACCACCAACTGCTTAG-3’, reverse 5’-GGTCATGAGTCCTTCCACGATAC-3’).

### Viability assay

**Fig 1:**
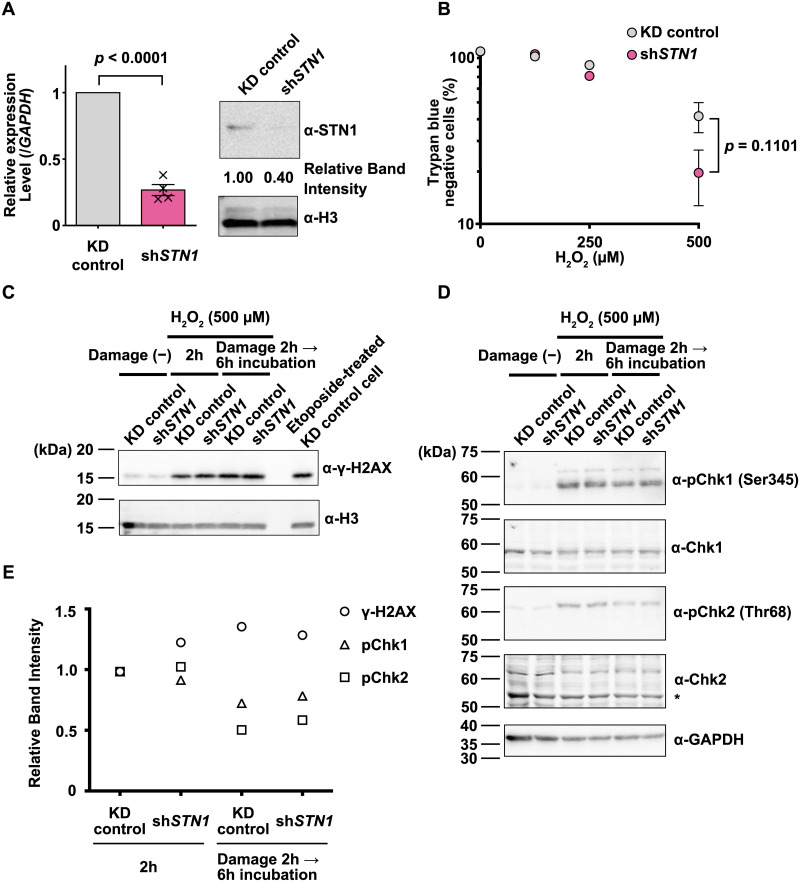
*STN1* suppression decreases cell viability upon hydrogen peroxide treatment. (A) Constitutive *STN1* knockdown in HeLa cells was evaluated by qRT-PCR and immunoblotting. *STN1* RNA levels were normalized to *GAPDH* RNA levels. X-axis, cell lines. Y-axis, relative level of *STN1* RNA normalized to the KD control. Mean ± SEM (n = 4). The *p*-value for an unpaired *t*-test is shown. Histone H3, a loading control. (B) Viability assay of HeLa cells treated with the indicated doses of H_2_O_2_ for 2 hours. Viability was measured 2 days after H_2_O_2_ treatment using the trypan blue staining method. Mean ± SEM (n = 3). Sub-lethal doses of H_2_O_2_ (125 or 250 μM) did not change the viability significantly among replicates, leading to an apparent lack of SEM bars. The *p*-value for an unpaired *t*-test is shown. (C) Immunoblotting experiments showing phosphorylated histone H2AX (γ-H2AX) in KD control and sh*STN1* cells treated with 500 μM H_2_O_2_ in DMEM for 2 hours or 25 μM Etoposide in DMEM for 1 hour. Histone H3 was used as a loading control. (D) Immunoblotting experiments showing phosphorylated Chk1 and Chk2. Cells were fixed at the indicated time points and subjected to immunoblotting experiments. GAPDH was used as a loading control. * non-specific bands. (E) Protein bands shown in (C) and (D) were quantified and normalized to the values for KD controls that were treated with H_2_O_2_ for 2 hours.

HeLa cells were plated on a 6-cm tissue culture dish at 2.5 x 10^5^ cells per dish and incubated for 2 days prior to H_2_O_2_ treatment (Fig 1B). Cells were exposed to H_2_O_2_ (0–500 μM) for 2 hours, and then incubated for 2 days without H_2_O_2_. Finally, both adherent and floating cells were harvested by trypsinization and centrifugation. The collected cells were resuspended in an equal volume of PBS containing 0.4% trypan blue. A Countess automated cell counter (Thermo Fisher Scientific) was used to quantify trypan blue positive and negative cells.

To measure cell viability after mimosine treatment (**Fig 4**), cells were seeded on a 10-cm tissue culture dish at 5 x 10^5^ cells (for mimosine treatment) or 2.5 x 10^5^ cells (for non-treatment control) per dish, incubated for 2 days, and then treated with mimosine (500 μM, final concentration) for 23 hours. Cells were either released to enter S phase by replacing the medium with fresh medium lacking mimosine, then were treated with H_2_O_2_, or they were treated with H_2_O_2_ in the presence of mimosine. Details are provided in the legend for **Fig 4A**.

To estimate cell viability by Propidium Iodide (PI) staining (S2 Fig), HeLa cells were plated on a 6-cm tissue culture dish and collected according to the protocol described above. The collected cells were resuspended in PBS to a concentration of 5 x 10^5^ cells/mL. After the addition of Propidium Iodide (PI, Nacalai) to a final concentration of 2 μg/mL, the cell suspensions were incubated for 15 min. Finally, PI fluorescence was analyzed using a BD Accuri C6 Plus Flowcytometer and FlowJoV10 software (BD Biosciences).

### Immunoblotting

HeLa cells were seeded on a 6-cm tissue culture dish at 2.5 x 10^5^ cells per dish, incubated for 2 days as described above, treated with genotoxic reagents (H_2_O_2_ or Etoposide) as indicated in each figure legend, and harvested by trypsinization and centrifugation (100×*g*, 4°C, 5 min).

For detection of cytoplasmic proteins (Chk1, Phospho-Chk1 (Ser345), Chk2, Phospho-Chk2 (Thr68) and α-Tubulin), the cells were treated with CSK buffer (10 mM PIPES (pH6.8), 300 mM sucrose, 150 mM NaCl, 3 mM MgCl_2_, 1 mM EGTA, 0.2 mg/mL phenylmethylsulfonyl fluoride (PMSF), 1 mM dithiothreitol (DTT)) containing 1x Complete EDTA free (Roche, Mannheim, Germany), PhosSTOP (Roche) and 0.1% (v/v) Triton X-100 [3] for 30 min on ice, followed by centrifugation (20,400×*g*, 4°C, 5 min). The protein concentration was determined with Protein Assay Dye Reagent Concentrate (Bio-Rad, Richmond, CA, United States), and adjusted to a standard concentration as necessary. The resultant protein extracts were denatured with 3 x SDS sample buffer (150 mM Tris-HCl (pH 6.8), 30% (v/v) glycerol, 6% (w/v) SDS, 18% (v/v) 2-mercaptoethanol, 0.01% (v/v) bromophenol blue) at 100°C for 5 min. For immunoblots of DNA binding proteins (RPA32, Phospho-RPA32 (S4/S8), STN1, Histone γ-H2AX and Histone H3), equal numbers of cells per sample were subjected to cell lysis with SDS lysis buffer (0.5% SDS, 10 mM HEPES (pH 8.5), 50 U Benzonase (Merck Millipore), and 2 mM MgCl_2_) [31] for 5 min on ice, followed by centrifugation (10,000×*g*, 4°C, 5 min). The supernatants were denatured with 3 x SDS sample buffer at 100°C for 5 min.

The resultant protein extracts were separated by SDS-PAGE, then transferred onto Immobilon-FL 0.45 μm pore polyvinylidene fluoride (PVDF) membranes (Merck Millipore). The membranes were then incubated with 3% skim milk (MEGMILK SNOW BRAND Co.,Ltd., Tokyo, Japan) in Tris-NaCl-Tween (TNT) buffer (0.1 M Tris-HCl (pH 7.5), 150 mM NaCl, 0.1% Tween 20) or BlockingOne-P (Nacalai) for 1 hour. The membranes then were washed with TNT buffer and incubated overnight at 4°C with primary antibodies diluted in Can Get Signal Immunoreaction Enhancer Solution 1 (TOYOBO, Osaka, Japan). The next day, the membranes were washed with TNT buffer at room temperature for five minutes, four times, and then incubated with secondary antibodies diluted in TNT buffer with 0.01% SDS for 1 hour at room temperature. Protein signals were detected using Chemi-Lumi One reagent (Nacalai) with FUSION Solo S (Vilber-Lourmat, Marne-la-Vallee, France) or by an ODYSSEY Infrared Imaging System (LI-COR, Lincoln, NE, United States). Signal intensities for protein bands were quantified with ImageJ (Fiji).

### Immunostaining

HeLa cells were grown on 15-mm coverslips (Matsunami Glass, Osaka, Japan) in 6-well plates at 2 x 10^5^ cells per well in DMEM and incubated for 2 days prior to H_2_O_2_ treatment. To enhance the adhesion of U2OS cells to coverslips, the glass surface was precoated with a 1% gelatin solution (FUJIFILM Wako Pure Chemical). After DNA damage induction, the cells were washed with PBS three times and fixed with fixation buffer (PBS containing 3% paraformaldehyde (PFA, Nacalai or FUJIFILM Wako Pure Chemical), 2% Sucrose, and 0.5% Triton X-100) for 30 min on ice [32]. The cells were then washed three times with PBS for five minutes at room temperature, permeabilized with 0.5% Triton X-100 in PBS for 5 min on ice, washed four times with PBS for five minutes at room temperature, then blocked with 2% BSA in PBS for 1 hour, followed by addition of the primary antibody. After washing three times, the cells were incubated with appropriate secondary antibodies. The primary and secondary antibodies were diluted in PBS containing 2% BSA, as indicated in S1 Table. The incubation was carried out for 1 hour at room temperature, avoiding light. Finally, nuclei were counterstained with 4-,6-diamidino-2-phenylindole (DAPI) in PBS, and the coverslips were mounted on a glass slide using VECTASHIELD Mounting Medium (Vector Laboratories, Burlingame, CA, United States). Images were captured with a DeltaVision Elite system (Cytiva, Marlborough, MA, United States) equipped with a 20x, 40x, or 60x objective lens. The images were processed by SoftWoRx (Cytiva).

To label newly synthesized DNA with 5-ethynyl-2’-deoxyuridine (EdU) or 5-bromo-2’-deoxyuridine (BrdU), EdU was dissolved in DMEM to a concentration of 10 μM and incorporated into proliferating cells on cover slips for the times indicated in each respective figure legend. Prior to DAPI-staining in the regular immunostaining procedure, Click-iT EdU staining was performed according to the manufacturer’s instructions (Thermo Fisher Scientific). For BrdU labelling, cells were cultured in media containing 20 μM BrdU for 10 min and then washed twice with PBS. After permeabilization following the previous immunostaining procedure, cells were rinsed with water, and then incubated with 2 M HCl at RT for 30 min to denature the DNA. Next, the denaturing buffer was neutralized with 1 M Tris base solution, and the samples were washed with PBS. The samples were treated with 1x BlockAce (Sumitomo Pharma Promo, Osaka, Japan) solution at RT for 30 min and then incubated overnight with anti-BrdU antibody (#BMC9318; Roche; 1:400) diluted in PBS containing 0.2% Tween20 at 4°C. After washing, the secondary anti-BrdU antibody in PBS containing 0.2% Tween20 was applied. DAPI-staining was carried out following the previous immunostaining procedure.

Signal intensities for EdU and RAD51 foci and the number of foci in fluorescence microscopy images were analyzed using a set of custom groovy scripts with ImageJ (Fiji). Briefly, DAPI images were segmented using a “Mexican Hat Filter” plug-in (https://imagej.nih.gov/ij/plugins/mexican-hat/index.html), and nuclei were identified by a built-in “Analyze Particles” function. After background subtraction (using a built-in “Subtract Background” function), mean fluorescence intensity for γ-H2AX or EdU within each nucleus was quantified. To identify RAD51 foci, a built-in “Find Maxima” function was used.

### Comet assay

Comet assays were carried out using a comet assay kit (Bio-Techne, Minneapolis, MN, United States) according to the manufacturer’s instructions. Briefly, HeLa cells were seeded on a 6-cm tissue culture dish at 2.5 x 10^5^ cells per dish and incubated for 2 days prior to H_2_O_2_ treatment. The cells were mixed with molten low-melting-point agarose and plated onto a comet slide. The slides were placed flat in a humid chamber at 4°C for gelation. For neutral comet assays, slides were incubated in the lysis solution for 2 hours at 4°C. Electrophoresis was carried out in an electrophoresis tank (BIO CRAFT, Tokyo, Japan) filled with the neutral electrophoresis buffer (100 mM Tris and 300 mM sodium acetate at pH 9.0) for 45 min at 4°C. The gels on the slides were then soaked in 7.5 M ammonium acetate and 95% ethanol, in that order, then dried at room temperature overnight. For alkaline comet assays, slides were incubated in the lysis solution (provided in the kit) overnight at 4°C. Electrophoresis was carried out in an electrophoresis tank filled with denaturing electrophoresis buffer (400 mM NaOH, 2 mM EDTA) for 30 min at 4°C. The gels were soaked in 70% ethanol, then dried on a heat plate set at 37°C. Finally, both types of samples, neutral and alkaline, were stained with SYBR Gold dye (Thermo Fisher Scientific). Images were captured with a DeltaVison Elite system (Cytiva) equipped with a 10x objective lens.

To analyze tail lengths of comets, ImageJ software was used. For alkaline comet assays, the OpenComet plug-in [33] was used. For neutral comet assays, the length was measured manually from the center of the head region to the end of the tail region, where the signal intensity reaches the background level along with the tail line.

### Flow cytometry analysis

Flow cytometric immunofluorescence assays were performed as previously described [34]. Briefly, HeLa cells were plated on two 10-cm tissue culture dishes at 5 x 10^5^ cells per dish and incubated for 3 days prior to DNA damage induction (H_2_O_2_ or HU). After exposure to the genotoxic reagents at concentrations and durations described in the figure legends, cells were harvested by trypsinization and fixed with PBS containing 1% PFA on ice for 15 minutes. The fixed cells were subsequently permeabilized in 80% ethanol at -20°C overnight. The cells were immunostained with anti-γ-H2AX antibody (for 2 hours) and Alexa 488-conjugated secondary antibody (for 30 min). The antibodies were diluted in PBS containing 1% BSA before use. Finally, genomic DNA was counterstained with 5 μg/mL Propidium Iodide (Nacalai), and RNA was degraded by incubation with 0.1 mg/mL RNaseA (Nacalai). The cells were then analyzed by BD FACSAria III and FlowJoV10 (BD Biosciences).

To analyze cell cycle progression (S6C Fig), HeLa cells were collected in 15 mL tubes at the indicated times after mimosine treatment according to the protocol described above (**Viability assay)**. The collected cell pellet was resuspended in 1 mL of PBS and fixed by adding 5 mL of 100% EtOH drop by drop while vortexing. The fixed cells were treated with 0.1 mg/mL RNaseA and stained with 50 μg/mL PI. The PI fluorescence signal was detected by a BD Accuri C6 Plus Flowcytometer and analyzed using FlowJoV10 software (BD Biosciences).

### Antibodies list

Antibodies and working dilutions used in this study are listed in S1 Table.

### Software and statistical analysis

Image processing was performed with Fiji (Fiji is just ImageJ v2.3.0/1.53f) [35]. Numerical data organization and calculations were performed with Microsoft Excel for Mac. Unpaired *t*-tests and Mann-Whitney *U* tests were carried out using GraphPad Prism software (version 6) for Mac OS (GraphPad Software, San Diego, CA, United States). The Anderson-Darling test was carried out using the ‘ad.test’ function in the ‘nortest’ R package (https://CRAN.R-project.org/package=nortest). Panels showing each result were generated using GraphPad Prism software. Figure panels were designed using Affinity Designer (v1.10.5). Source codes for image analyses and statistical tests are available at: https://github.com/HidenoriNakaoka/Hara_et_al.

## Results

### STN1 is required for survival under high levels of oxidative stress

It is well known that H_2_O_2_ treatment directly induces various types of DNA damage (including DSBs and SSBs) in cells [36, 37]. To investigate the role of STN1 in cells undergoing DNA strand breaks, we generated a constitutive *STN1* knockdown (KD) HeLa cell line (sh*STN1* cells, Fig 1A) and treated the cells with H_2_O_2_. Cells transduced with an empty lentiviral vector were used as control cells (KD control). Cells were treated for 2 hours with 0, 125, 250 or 500 μM H_2_O_2_, and their viability was evaluated by trypan blue staining two days after the treatment. After exposure to 500 μM H_2_O_2_, sh*STN1* cells showed a lower viability compared with KD control cells (Fig 1B. See also S1 Fig for another shSTN1 construct. The viability assay by PI staining is described in S2 Fig), suggesting that STN1 is required for cellular survival under oxidative stress. All three subunits, CTC1, STN1, and TEN1, are essential for the CST complex to accomplish its various functions [23, 28, 38, 39]. Indeed, we confirmed that *CTC1* knockdown cells exhibited a similar level of sensitivity to H_2_O_2_, supporting the notion that the reduced viability is caused by the loss of the functional CST trimeric complex (S1D and S1E Fig).

Next, to examine if *STN1* KD affects the level of DNA damage(s) and subsequent DNA damage checkpoint responses, we first monitored phosphorylation of Ser139 of Histone H2AX (γ-H2AX) by immunoblotting (Fig 1C and 1E) [reviewed in 40]. An etoposide (topoisomerase II inhibitor)-treated sample was used as a positive control. After 2 hours of 500 μM H_2_O_2_ treatment, the γ-H2AX signal was similarly increased in both KD control and sh*STN1* cells. We also found that a six hour-recovery after the addition of H_2_O_2_ did not reduce the γ-H2AX signal in both lines. Similarly, phosphorylation of both Chk1 (Ser345) and Chk2 (Thr68) in H_2_O_2_-treated KD control and sh*STN1* cells persisted 6 hours after H_2_O_2_ treatment, as was observed with the γ-H2AX signal (Fig 1D and 1E). These results suggested that the absence of STN1 did not impede the DNA damage checkpoint signaling cascades.

We next conducted indirect IF staining of γ-H2AX to observe DNA damage accumulation in individual cells (S3 Fig). Consistent with previous reports, H_2_O_2_-induced γ-H2AX signals appeared not to be sharply defined, but rather non-homogeneous blobs in the nucleus, unlike discrete foci induced by ionizing radiation [41]. We found that essentially all H_2_O_2_-treated cells showed positive γ-H2AX signals in the nuclei, suggesting that DNA damage induction by H_2_O_2_ is not restricted to a specific cell cycle stage. *STN1* KD did not alter the intensity and spatial distribution of γ-H2AX signals in nuclei.

**Fig 2:**
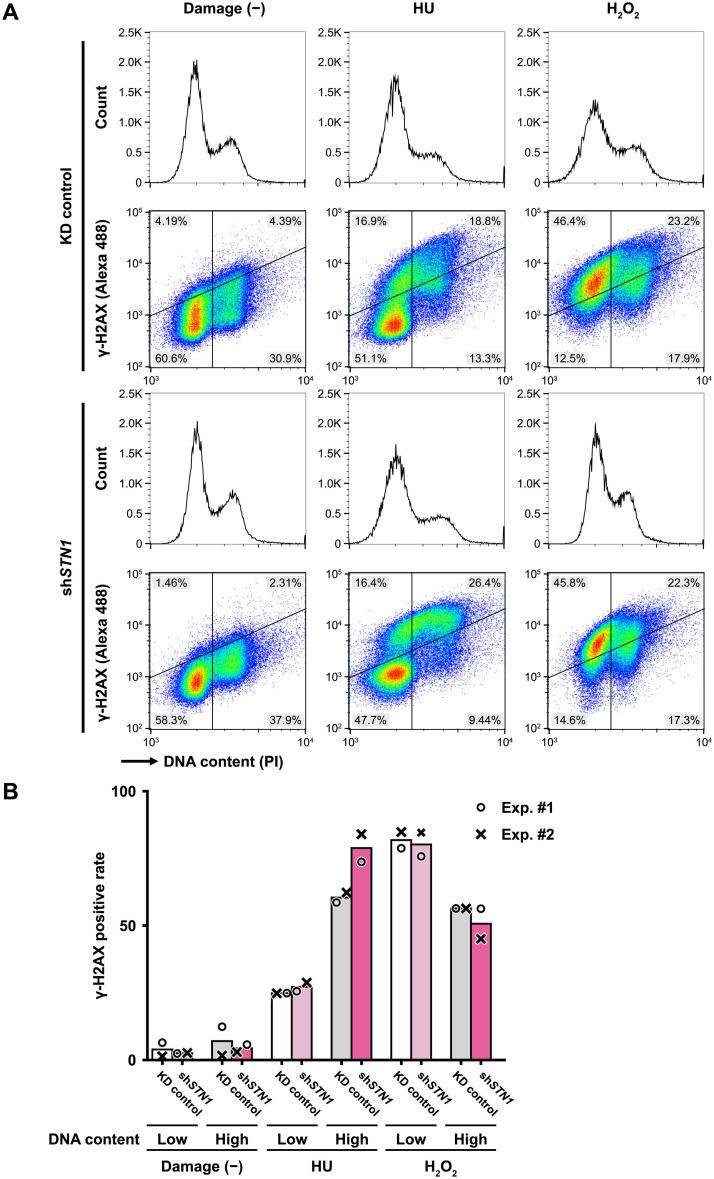
Flow cytometric analyses of γ-H2AX levels in HU- or H_2_O_2_-treated HeLa cells. (A) HeLa cells were treated with either HU (5 μM, 3 hours) or H_2_O_2_ (500 μM, 10 min (See S4C Fig for details)) and immediately fixed with 1% PFA. The fixed cells were double-stained with PI (for DNA content) and Alexa488-conjugated anti-γ-H2AX antibody, and subjected to flow cytometry. The horizontal and vertical axes of density scatter plots represent PI fluorescence and Alexa488 fluorescence, respectively. Two lines that divide the density scatter plots into four orthants (1^st^ orthant, high DNA content, high γ-H2AX level; 2^nd^ orthant, low DNA content, high γ-H2AX level; 3^rd^ orthant, low DNA content, low γ-H2AX level; 4^th^ orthant, high DNA content, low γ-H2AX level) were manually determined on the plot for Damage (-) KD control, and the same lines were applied to the other plots. Percentages of each sub-population are shown at the corners of the density plots. A 1D-histogram for PI fluorescence distribution is shown above each scatter plot. The vertical axis indicates the normalized (modal normalization) cell count. PI fluorescence signal values in each panel were adjusted so that the signal intensities for G1 peaks are the same among experiments. (B) γ-H2AX positive rates (percentage) for low-DNA content populations (*f*_2_/(*f*_2_ + *f*_3_)) and high-DNA content populations (*f*_1_/(*f*_1_ + *f*_4_)) were computed, where *f*_*i*_ is a population fraction for the *i*-th orthant. Exp. #1 and #2 refer to sets of experiments shown in Fig 2A and S4 Fig, respectively.

Cells treated with HU or H_2_O_2_ along with untreated cells were stained with both PI and an Alexa488-conjugated antibody against γ-H2AX, and then subjected to flow cytometry [34, 42] (Fig 2A, S4A and S4B Fig). Untreated KD control and sh*STN1* cells showed similar profiles of γ-H2AX signals, which are interpreted as basal levels (Fig 2A, left column; S4A Fig, left panel). As expected, HU treatment led to enhanced γ-H2AX signals [42–44] (Fig 2A and S4B Fig, middle columns). While approximately 60–70% of the high-DNA content populations exhibited an upshift of the γ-H2AX signal, only 25% of the low-DNA content populations did so, most likely due to the insensitivity of G1 cells to HU (Fig 2B). We noticed that the distribution of γ-H2AX fluorescence for HU-treated sh*STN1* cells was right-shifted relative to that of KD control cells (S4A Fig, middle panel). Furthermore, the fractions of γ-H2AX positive cells in high-DNA content populations were slightly higher for HU-treated sh*STN1* cells than KD control (Fig 2B). Our observation of an augmented HU-induced γ-H2AX response in sh*STN1* cells is consistent with a current view that CST is required for restarting stalled replication forks [23, 29].

Contrasting with the results for HU treatment, when cells were treated with H_2_O_2_, the entire cell population showed an increase in γ-H2AX intensity (compare left and right columns in Fig 2A and 2B, S4B Fig). These results suggest that the H_2_O_2_ treatment induced γ-H2AX-positive cells similarly throughout the cell cycle, which is consistent with the anti-γ-H2AX IF results shown in S3 Fig. Moreover, the distribution of γ-H2AX signal for H_2_O_2_ treated sh*STN1* cells was almost identical to that of control KD cells (S4A Fig, right panel), suggesting that the signaling cascade in the DNA damage checkpoint that culminates in the phosphorylation of H2AX was unaffected by the absence of STN1.

### Increased DNA strand breaks are observed in H_2_O_2_-treated sh*STN1* cells

**Fig 3:**
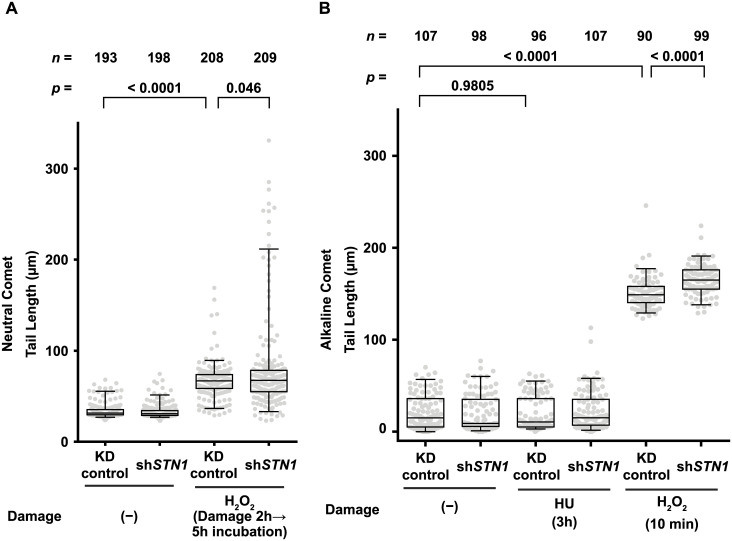
*STN1* KD exacerbates DNA damage induced by H_2_O_2_. (A) Box-and-whisker plots showing distributions of neutral comet tail lengths for each experimental condition. Cells were treated with 500 μM H_2_O_2_ in DMEM for 2 hours, followed by 5 hours incubation in DMEM, then were subjected to a neutral comet assay (see Materials and methods for details). Numbers of analyzed comets (*n*) are shown above each lane. *p*-values for Anderson-Darling tests are shown above respective paired lanes. (B) Box-and-whisker plots showing distributions of alkaline comet tail lengths for each experimental condition. Prior to the assay, cells were treated with 500 μM H_2_O_2_ in DMEM for 10 minutes or 5 μM HU in DMEM for 3 hours. The apparent lack of DNA strand breaks in the HU-treated cells does not necessarily contradict the flow cytometry data shown in Fig 2: histone H2AX can be phosphorylated by ATR, which is activated by exposed ssDNA within stalled replication forks [45]. Tail lengths were measured by the “OpenComet” plugin in ImageJ software [35]. For each condition, numbers of analyzed comets (*n*) are shown above each lane. *p*-values for Mann–Whitney *U* tests are shown above respective paired lanes.

Although the DNA damage and checkpoint markers tested above did not reveal significant differences between H_2_O_2_-treated KD control and sh*STN1* cells, it was still possible that larger amounts of DNA strand breaks were generated in sh*STN1* cells than in KD control cells upon H_2_O_2_ treatment. To test this, we next performed comet assays in both neutral and alkaline conditions to detect DSBs and SSBs. Comet tail lengths in neutral conditions were not affected by sh*STN1* in the absence of DNA damaging agents (median length ~ 30 μm) (Fig 3A). As expected, H_2_O_2_ treatment increased median comet tail lengths (median length ~ 65 μm) regardless of *STN1* knockdown, indicating the generation of DSBs by H_2_O_2_. Importantly, however, the distribution of the comet tail length for H_2_O_2_-treated sh*STN1* cells had a longer tail than that of the KD control, which was statistically significant (*p* = 0.046 for the 2-sample Anderson-Darling test) (Fig 3A. See also S5A and S5C Fig), suggesting that a fraction of sh*STN1* cells experience extensive DSBs upon H_2_O_2_ treatment. The extensive DNA strand breaks in sh*STN1* cells were further confirmed by an alkaline comet assay (Fig 3B): while HU treatment did not produce noticeable changes in the distributions of the comet tail lengths, H_2_O_2_ treatment significantly increased the lengths of the comet tails. Moreover, the median comet tail length for sh*STN1* cells was longer than that for the KD control (*p* < 0.0001 for the Mann-Whitney *U* test) (Fig 3B See also S5B and S5D Fig). Taken together, these data suggest that DNA strand breaks are induced more frequently and/or less efficiently repaired in sh*STN1* cells than in KD control cells upon H_2_O_2_ treatment.

### sh*STN1* cells are vulnerable to H_2_O_2_ in S phase

**Fig 4:**
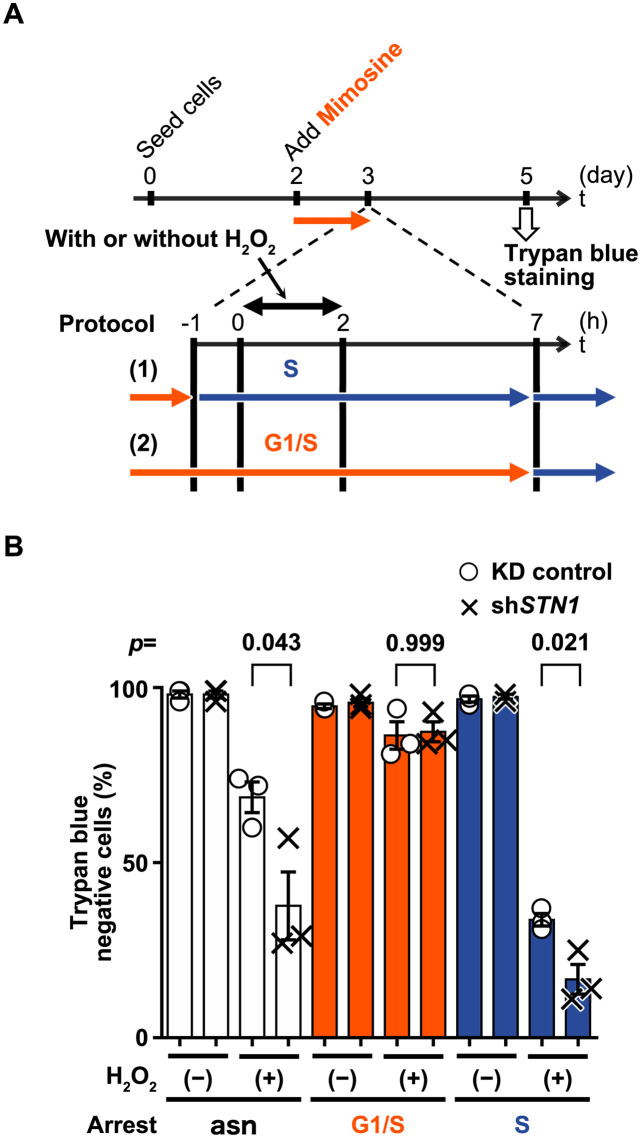
S phase but not G1/S-arrested sh*STN1* cells are sensitive to H_2_O_2_. (A) Experimental design. The cell cycle was arrested at the G1/S boundary by mimosine treatment for 23 hours. Protocol (1): for the damage induction in S phase, the G1/S-arrested cells were released into S phase by removing mimosine one hour before the H_2_O_2_ treatment (the one-hour delay is required to guarantee the entry into S phase. See S6 Fig for a timeline.). Protocol (2): the mimosine-arrested cells were exposed to H_2_O_2_-containing (500 μM) DMEM for 2 hours. After the removal of H_2_O_2_, G1/S arrest was maintained for a further 5 hours with mimosine, after which mimosine was removed by washing with PBS, then replacing the medium with fresh medium lacking mimosine. In both protocols, viability was measured by trypan blue staining two days after the damage induction. (B) Viabilities of H_2_O_2_-treated HeLa cells at *t* = 5 days. *p*-values for unpaired *t*-tests are shown above respective paired bars. asn, asynchronous (without mimosine treatment); G1, cells under mimosine arrest; S, cells that were released from mimosine arrest. Data from three biological replicates are shown with mean values and SEM.

Although H_2_O_2_ damages DNA independently of cell cycle stages (Fig 2), the observed loss of viability in sh*STN1* cells (Fig 1B) could be caused by failures in cell-cycle-specific events such as DNA replication. To test this hypothesis, we compared the H_2_O_2_ sensitivities of asynchronous (asn), G1/S-arrested, and S-phase synchronized populations of sh*STN1* cells along with their KD control counterparts (Fig 4A and 4B. See also S6 Fig for validation of the cell-cycle synchronization). Cells arrested at the G1/S boundary by mimosine treatment for ~24 hours were released into S phase (Fig 4A, Protocol (1)) or maintained in the arrested state (Fig 4A, Protocol (2)), and then exposed to H_2_O_2_-containing medium (H_2_O_2_ (+)) or standard medium (H_2_O_2_ (-)) for 2 hours. After 2 days of recovery, the viability of each population was evaluated by trypan blue staining (Fig 4B). In the absence of H_2_O_2_ treatment, all three populations (asn, G1/S, and S) of both sh*STN1* and KD control cells were virtually 100% viable, indicating that mimosine treatment by itself did not affect viability (Fig 4B, H_2_O_2_ (-)). For the asynchronous populations treated with H_2_O_2_, mean viabilities decreased significantly to 69% and 38% for KD control and sh*STN1* cells, respectively (Fig 4B, white bars), which is consistent with the results shown in Fig 1B. Interestingly, we found that G1/S-arrested populations of both sh*STN1* and KD control cells were relatively insensitive to H_2_O_2_: 86% viable for the KD control, and 87% viable for sh*STN1* cells (Fig 4B, red bars, H_2_O_2_ (+)). In contrast, S-phase populations were more sensitive to H_2_O_2_ treatment than the asynchronous populations: 34% viable for the KD control and 17% viable for sh*STN1* cells (Fig 4B, blue bars, H_2_O_2_ (+)). These results suggest that HeLa cells are sensitive to H_2_O_2_ particularly in S phase, and that the knockdown of *STN1* exacerbated the sensitivity.

### sh*STN1* cells in S phase fail to resume DNA synthesis after H_2_O_2_ treatment

**Fig 5:**
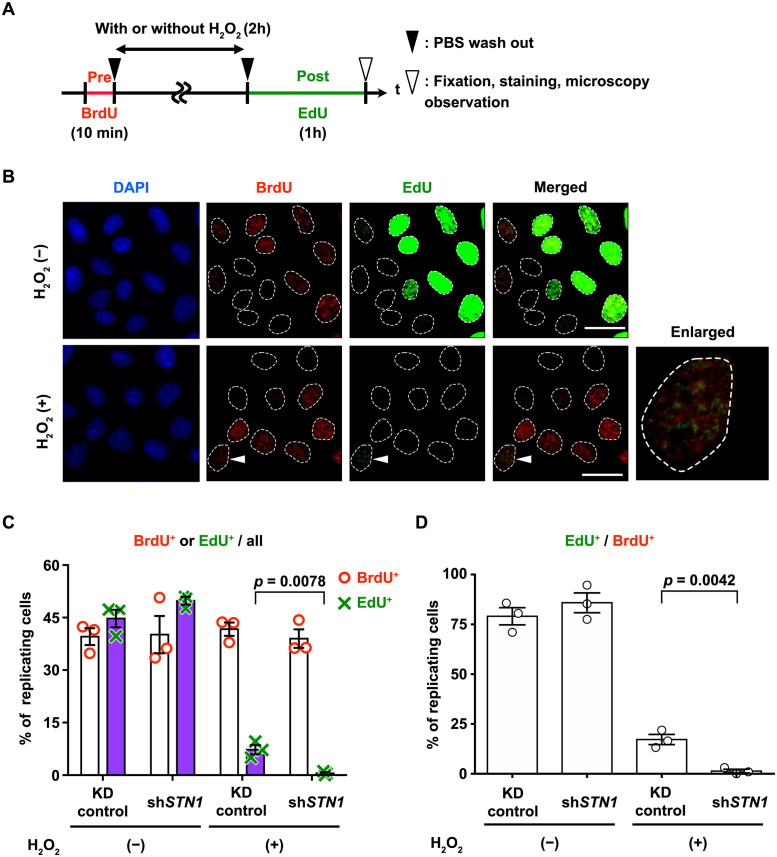
H_2_O_2_ -treated sh*STN1* cells fail to resume DNA synthesis in S phase. (A) Experimental design. Asynchronous cell populations were pulse-labeled with BrdU prior to H_2_O_2_ treatment (500 μM, 2 hours. The mock-treated cells were allowed to grow for 2 hours.). Immediately after washing out the H_2_O_2_, the cells were allowed to incorporate EdU for one hour. (B) Representative microscopic images for KD control cells. DAPI staining was used to identify nuclei. The merged column shows superpositions of BrdU (detected by Cy5-conjugated antibody) and EdU (detected by conjugated Alexa Fluor 488) signals. Top row: Mock-treated cells. The majority of the BrdU-positive nuclei are also EdU-positive, indicating unperturbed progression of chromosomal replication. Bottom row: H_2_O_2_-treated cells. The majority of the BrdU-positive nuclei are EdU-negative, indicating suppression of DNA replication upon H_2_O_2_ treatment. An example of a BrdU/EdU-double-positive nucleus is indicated by a white arrowhead, and its enlarged image is shown in the rightmost column. Scale bars, 20 μm. (C) Percentage of BrdU or EdU positive cells in each condition. Data from three biological replicates are shown with mean values (boxes) and SEM. (D) Percentages of EdU-positive cells among BrdU-positive cells. Data from three biological replicates are shown with mean values (boxes) and SEM. For (C) and (D), at least 200 cells per condition were randomly selected in each assay. *p*-values for unpaired *t*-tests are shown above respective paired bars.

A simple explanation for the above observation would be that DNA strand breaks induced during chromosomal replication are highly detrimental, and the CST complex plays some role in coping with such situations. To investigate whether the CST complex functions in the resumption of the perturbed replication forks in H_2_O_2_-treated cells, we assayed nucleotide incorporation activities of the S-phase cells after H_2_O_2_ treatment (Fig 5A). To identify S-phase cells in asynchronous sh*STN1* or KD control populations, cells were pulse-labeled with BrdU prior to H_2_O_2_ treatment. Note that in all cell populations tested, the fraction of S phase (i.e. BrdU-positive) cells was approx. 40% (Fig 5C, white bars). Subsequently, the cells were exposed to H_2_O_2_-containing medium (H_2_O_2_ (+)) or standard medium (H_2_O_2_ (-)) for 2 hours, and then cultured for 1 hour in EdU-containing medium. After fixation and staining with a Cy5-conjugated antibody (for BrdU) and an Alexa Fluor 488 azide (for EdU), incorporation of the nucleotide analogues was detected by fluorescence microscopy (Fig 5B–5D).

In mock-treated sh*STN1* and KD control cells (H_2_O_2_ (-)), most of the BrdU-positive cells were also EdU-positive, as expected (Fig 5B top row, and Fig 5D). In H_2_O_2_-treated cell populations, however, the fractions of EdU-positive nuclei significantly decreased. While 7% (56/756 cells in total) of H_2_O_2_-treated KD control cells incorporated EdU within 1 hour following the completion of H_2_O_2_ treatment, only 0.5% (4/865 cells in total) of H_2_O_2_-treated sh*STN1* cells were EdU-positive (Fig 5C, purple bars, H_2_O_2_ (+)). Focusing on S-phase (BrdU-positive) cells, 17% (56/316 cells) and 1.2% (4/340 cells) of KD control cells and sh*STN1* cells, respectively, incorporated EdU (Fig 5D, H_2_O_2_ (+)). These results suggested that DNA replication largely halted after H_2_O_2_ treatment and that sh*STN1* cells almost completely ceased to incorporate nucleotides.

We noticed that the EdU signal intensity in the H_2_O_2_-treated nuclei was much lower than that in the mock-treated condition (Fig 5B, S7 Fig). This formally raises the possibility that the EdU incorporation is due to the synthesis of short stretches of DNA in, for example, nucleotide excision repair and/or long-patch base excision repair, which can occur independently of DNA replication. It is of note, however, that we did not observe EdU incorporation outside of S phase; all EdU-positive nuclei were also BrdU-positive. Therefore, it is most likely that the EdU incorporation is associated with some form of resumption process in the perturbed replication forks in S phase.

In summary, these results suggest that the loss of viability after the H_2_O_2_ treatment was caused by failure to complete DNA replication in S phase, and the presence of the CST complex increased the probability of successful recovery from the H_2_O_2_-induced DNA damage during S phase.

### RAD51 nuclear foci decrease in response to oxidative damage in sh*STN1* cells

**Fig 6:**
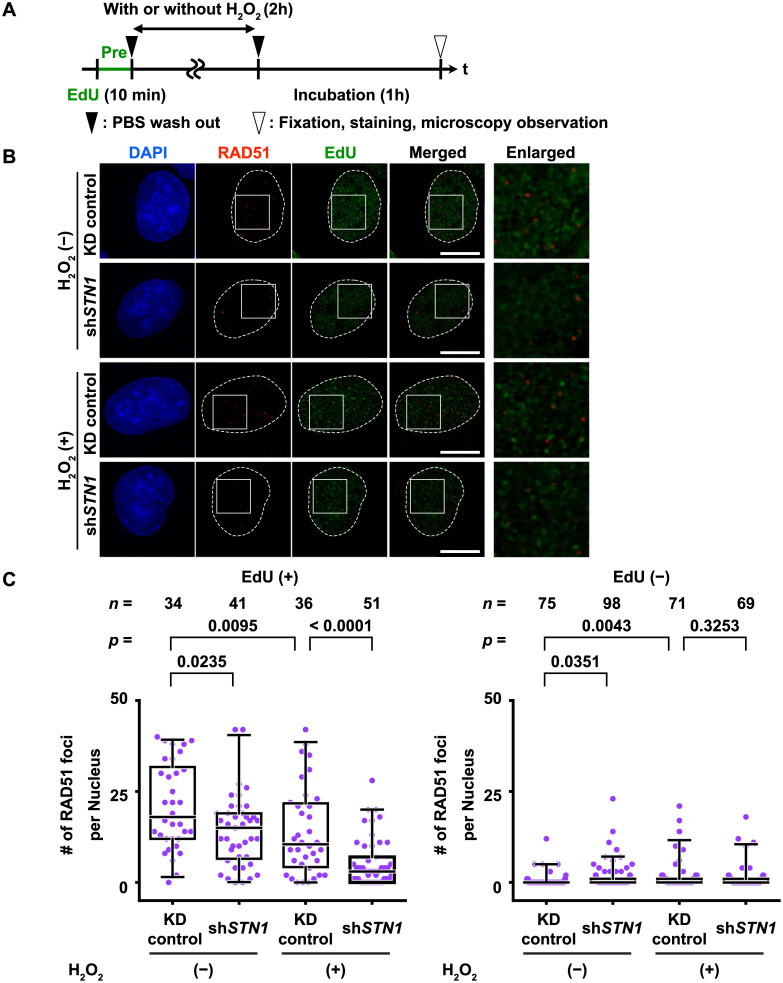
STN1 is required for nuclear RAD51 foci formation after oxidative damage. (A) The experimental timeline. Cells in S phase were pulse-labeled with EdU prior to H_2_O_2_ treatment (500 μM, 2 hours). After a one-hour recovery, the cells were fixed and immuno-stained with anti-RAD51 antibody. (B) Representative images of RAD51 foci in an EdU-positive nucleus. The enlarged area shown in the rightmost column is indicated by white squares. Scale bar: 10 μm. (C) Box-and-whisker plots showing the number of nuclear RAD51 foci per nucleus (Y-axis). At least 50 nuclei were randomly selected in each experimental condition. Results from two independent experiments were combined (individual data for the two experiments are shown in S8 Fig). Numbers of analyzed nuclei (*n*) are shown at the top of each lane. *p*-values for Mann–Whitney *U* tests are shown above respective paired lanes. See also S9 Fig for the same experiments using U2OS cells.

One plausible explanation for the failure of DNA synthesis in the H_2_O_2_-treated cells is that DNA strand breaks generated by H_2_O_2_ (and/or subsequent replication fork collapse) remain unrepaired, causing prolonged activation of the DNA damage checkpoint. As RAD51 is a key molecule that deals with DNA lesions induced by replication stress (e.g., promotion of strand invasion for HR-dependent DSB repair or break-induced replication, and protection of exposed ssDNA from nucleolytic attack [25]), we asked if RAD51 nuclear foci formation is compromised in sh*STN1* HeLa cells in S phase. EdU pulse-labeling was carried out immediately before H_2_O_2_ treatment to distinguish S phase cells, and RAD51 nuclear foci were detected by indirect immunofluorescence microscopy (Fig 6A and 6B). In mock-treated cells, several to several tens of RAD51 foci per nucleus were observed, which presumably represents background levels of DNA damage response in the unstressed culture conditions (Fig 6B and 6C, H_2_O_2_ (-)). We detected RAD51 foci in H_2_O_2_-treated KD control cells, albeit fewer compared to the untreated cases. These RAD51 foci did not co-localize with EdU foci, suggesting that the H_2_O_2_-induced DNA damage sites did not coincide with the replicating sites at the beginning of the experimental timeline (Fig 6B, Enlarged). In H_2_O_2_-treated sh*STN1* cells, however, RAD51 foci formation was almost completely abolished (Fig 6C, H_2_O_2_ (+)). Essentially no RAD51 foci were observed in EdU-negative (i.e. cells outside of S phase) nuclei (Fig 6C, right panel). Collectively, our observations imply that STN1 is required to recruit RAD51 to DNA damage sites and/or to prevent exposed ssDNA, which is bound by RAD51, from being degraded.

### STN1 and RAD51 function in the same pathway to ensure survival of H_2_O_2_-treated cells

**Fig 7:**
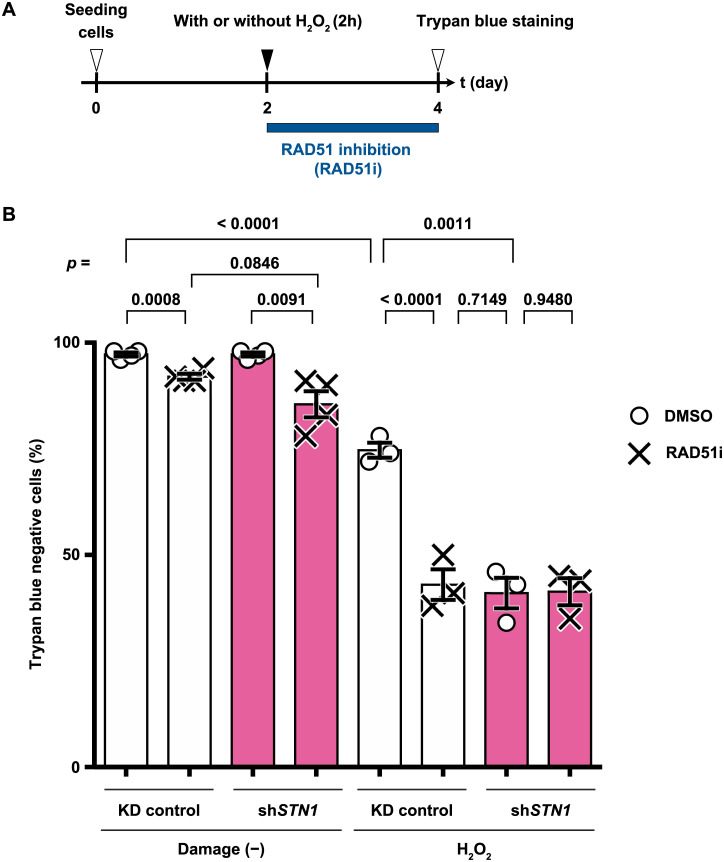
CST is an upstream factor of RAD51. (A) The experimental timeline. HeLa cells treated with H_2_O_2_ (500 μM, 2 hours) were then incubated for 2 days with DMEM containing RAD51 inhibitor B02 (20 μM final concentration) or the same volume of DMSO as a control. (B) Viabilities of H_2_O_2_-treated HeLa cells at *t* = 4 days measured by trypan blue staining. *p*-values for unpaired *t*-tests are shown above respective paired bars. Data from three or four biological replicates are shown with mean values (boxes) and SEM.

Immunostaining of RAD51 revealed that CST was required for the localization of RAD51 in the nuclei of the cells subjected to H_2_O_2_ treatment. To test whether the absence of RAD51 is sufficient to explain the loss of viability in H_2_O_2_-treated sh*STN1* cells, we chemically inhibited RAD51 in sh*STN1* or KD control cells and evaluated viabilities after H_2_O_2_ treatment (Fig 7A). B02 is a drug that inhibits RAD51 filament formation on ssDNA and subsequent DNA strand exchange (S10 Fig) [46, 47]. B02 treatment alone in KD control cells marginally affected viability (Fig 7B). When combined with H_2_O_2_ treatment, however, the viability of the KD control cells significantly decreased to less than 50%, which was similar to that of H_2_O_2_-treated sh*STN1* cells (Fig 7B). This indicates that the loss of RAD51 DNA binding activity and sh*STN1* have a similar impact on cellular survival upon H_2_O_2_ treatment. Importantly, B02 treatment did not exacerbate the sensitivity of sh*STN1* cells to H_2_O_2_, suggesting that RAD51 and STN1 function in the same pathway in response to H_2_O_2_-induced DNA damage (Fig 7B).

## Discussion

In this work, we first demonstrated that depletion of the CST complex sensitized cells to H_2_O_2_, an oxidizing reagent that induces DNA strand breaks. Although H_2_O_2_ induces DNA damage in a cell cycle-independent manner, we have revealed that CST is particularly required for maintaining viability in S-phase cells. We further demonstrated that CST-defective cells tend to abort DNA synthesis and fail to recruit homologous recombinational repair protein RAD51 to presumptive sites of DSBs. Below, we discuss molecular mechanisms of the S phase-specific role of CST in the response of cells to oxidative DNA damage.

H_2_O_2_ can induce both SSBs and DSBs according to the dose [37]. A relatively high concentration (0.5 mM) of H_2_O_2_, as used in our experiments, induces DSBs as well as SSBs. Indeed, we observed persistent γ-H2AX and DNA damage checkpoint activation after the H_2_O_2_ treatment (Fig 1C and 1D). We demonstrated that those DSBs were induced throughout the cell cycle, as we observed that: 1) virtually all cells became γ-H2AX-positive upon H_2_O_2_ treatment (Fig 2, S1C Fig); and 2) the majority of the nuclei exhibited comet tails in the neutral comet assay (Fig 3). This cell-cycle-independent mechanism of DNA strand break induction allowed us to investigate which particular phase of the cell cycle, if any, requires CST for processing/repairing the lesions (Fig 4). We found that sh*STN1* cells synchronized at G1 phase by mimosine treatment were insensitive to the high dose of H_2_O_2_ (Fig 4). Given that BER (base excision repair) and NHEJ (non-homologous end joining), rather than HR (homologous recombinational repair), are dominant DNA repair mechanisms in G1 phase [36, 48], our results suggest that the CST complex is not essential for non-HR DNA repair. On the other hand, sh*STN1* cells in S phase were highly sensitive to H_2_O_2_ (Fig 4). The CST complex is suggested to promote firing of dormant/late origins by recruiting or stimulating DNA Pol α in response to replication fork stalling [19, 22]. The same mechanism could be required for the recovery from oxidative stress in S phase. The suppression of new origin firing, however, could be a mere consequence of DNA damage checkpoint activation; the checkpoint kinases inhibit pre-initiation complex formation through degradation of CDC25 phosphatase [49]. Prolonged activation (phosphorylation) of Chk1 and Chk2 kinases is consistent with the latter hypothesis (Fig 1D).

Chastain *et al*. showed that the CST complex recruits RAD51 to stalled replication forks through an ATR (i.e. DNA damage checkpoint)-dependent physical interaction [23]. Another *in vitro* study also reported a physical interaction between the CST complex and RAD51, which was needed for loading RAD51 onto ssDNA [28]. Our observation that the accumulation of RAD51 onto chromatin after the H_2_O_2_ treatment was abolished in sh*STN1* cells is in line with the proposed function of the CST complex as a RAD51 loader (Figs 6 and 7). Besides the assistance in RAD51 loading, however, the CST complex might play a role in protecting the exposed ssDNA from further degradation by nucleases such as MRE11 [29]. Given that DSBs were extensively generated by the high dose of H_2_O_2_ treatment, and that RAD51 is best characterized as a homologous recombination protein required to repair such DNA lesions, the cell-cycle dependent sensitivity to H_2_O_2_ in sh*STN1* cells (Fig 4) could at least partly be explained by the deficiency in HR-dependent DSB repair in S phase. It should be stressed, however, that RAD51 is also involved in HR-independent mechanisms of replication stress responses: it binds to the exposed ssDNA regions in stalled replication forks and promotes fork regression, which is believed to be protective against nucleolytic degradation, followed by fork restart [reviewed in 50]. As H_2_O_2_ generates various types of DNA damage that can culminate in fork stalling in S phase, our results are also compatible with a model in which CST is required for fork restart in the presence of oxidative stress through the regulation of RAD51 loading onto the stalled forks.

Taken together, our work provides supportive evidence for the involvement of the CST complex in RAD51-dependent DNA repair/protection mechanisms in S phase (S11 Fig). We have not addressed the question of whether the CST complex plays a role in SSB repair or not, which should be investigated in a future separate study. It is of note, however, that while our manuscript was in preparation, STN1 deficiency was reported to down-regulate DNA glycosylases and thus suppress base excision repair (BER), leading to an increased oxidative DNA damage and risk of colon cancer [51]. Therefore, the CST complex might regulate different DNA repair mechanisms depending on the sources and doses of the oxidative stress and possibly cell types. Last but not least, it is interesting to note that the CST complex can enhance NHEJ rather than HR by promoting DNA Pol α-mediated fill-in synthesis at DSB sites, thereby preventing RAD51 from binding to the resected ends in BRCA1-deficient cells (i.e. HR-deficient cells) [52, 53]. Therefore, the CST complex likely has two opposite regulatory roles for RAD51 loading: 1) counteracting DSB resection by recruiting Pol α and thus preventing RAD51 loading and 2) promoting RAD51 filament formation at the resected or exposed ssDNA regions. The regulation of RAD51 by the CST complex is likely dependent on contexts such as cell type, genetic background, genotoxic reagents used to induce DNA damage, and cell cycle. How the CST complex, the RPA complex, RAD51, and other HR- or replication-related proteins are coordinated at the various types of DNA damage sites to facilitate repair is still unclear, and further investigation is needed to understand the detailed molecular mechanisms.

## Conclusions

We found that sh*STN1* cells were sensitized to H_2_O_2_ treatment, and observed that CST contributes to H_2_O_2_ resistance in an S phase-dependent manner. *In vivo*, both exogenous and endogenous ROS potentially cause DNA damage. Before it becomes too late for repair, a mechanism for rapid restoration of the damaged genome is considered to be important. Our study provides insights into how the CST complex mediates the cellular response to oxidation-induced DNA strand breaks.

## Supporting information

S1 Fig Knockdown of CST components impairs cell viability during hydrogen peroxide challenge. (A) Growth curve of HeLa or U2OS sh*STN1* cells. (B) Constitutive *STN1* knockdown in HeLa cells using the sh*STN1*#2 construct was evaluated by qRT-PCR. *STN1* RNA levels were normalized to *GAPDH* RNA levels. X-axis, cell lines. Y-axis, relative level of *STN1* RNA normalized to the KD control. Error-bars represent SEM. (C) Viability assay of HeLa cells treated with 500 μM of H_2_O_2_ for 2 hours. Viability was measured 2 days after the H_2_O_2_ treatment by trypan blue staining. Data from three biological replicates are shown with mean values (boxes). The *p*-value for the unpaired *t*-test is shown above the indicated bars. (D) Constitutive *CTC1* knockdown in HeLa cells was evaluated by qRT-PCR. *CTC1* RNA levels were normalized to *GAPDH* RNA levels. X-axis, cell lines. Y-axis, relative level of *CTC1* RNA normalized to the KD control. Mean ± SEM (n = 4). (E) Viability assay of HeLa cells treated with the indicated doses of H_2_O_2_ for 2 hours. Error-bars represent SEM. The *p*-value for the unpaired *t*-test is shown. We note that it was previously reported that lack of any CST component results in unstable binding between CST components and ssDNA [3, 54]. Thus, in this research, we preferentially used cultured cells depleted of STN1, which bridges CTC1 and TEN1.
(PDF)

S2 Fig Trypan blue and PI staining give comparable estimates of cell viability. (A) Experimental design. HeLa cells were treated with H_2_O_2_ (500 μM, 2 hours). Immediately after washing out the H_2_O_2_, the cells were incubated for two days in standard medium. Finally, all cells were collected, and half of the sample was subjected to Trypan blue staining, while the other half was subjected to PI staining. (B) Representative data set of flow cytometric analysis of PI-stained cells. Left-side panels are representative forward scatter versus side scatter dot plots, showing 10,000 cells in each panel. The square gates at the bottom-left corners show debris that was excluded from the analysis. The red dots are PI-positive cells, which were determined according to the histograms shown in the right side. In the histograms, blue-colored regions indicate PI-negative fractions. (C) Representative images of trypan-blue-stained cells taken by a Countess automated cell counter. Bright-field images were automatically processed by the equipment software to identify stained (dead) and unstained (alive) cells. In the processed images, dead and live cells are indicated by red and green, respectively. (D) Comparison between trypan blue staining and PI staining. In each experimental condition, data points for the estimated viabilities with the two methods are connected by broken lines. Data from three biological replicates are shown with mean values (boxes). *p*-values for unpaired *t*-tests are shown above the respective paired bars.
(PDF)

S3 Fig *STN1* KD increased γ-H2AX signal intensity in both HU- and H_2_O_2_-treated HeLa cells. (A) Representative images of an indirect immunofluorescence assay for γ-H2AX in untreated, HU-, or H_2_O_2_-treated HeLa cells. Broken lines show nuclei of untreated or HU-treated cells. Scale bars, 20 μm. (B) Box-and-whisker plots showing the signal intensity of γ-H2AX (i.e. fluorescence intensity of Alexa Fluor 488 in a.u. [arbitrary units]) in the nuclei. In each condition, the signal intensities of 800 randomly selected nuclei are shown. *p*-values for unpaired *t*-tests are shown above the respective bars.
(PDF)

S4 Fig Flow cytometric analyses of γ-H2AX levels in HU- or H_2_O_2_-treated HeLa cells. (A) Histograms of Alexa488 intensity in undamaged, HU- or H_2_O_2_-treated HeLa cells. The horizontal axis shows Alexa488 fluorescence (γ-H2AX level), and the vertical axis indicates cell counts for each intensity value. Blue-colored regions indicate γ-H2AX positive fractions. (B) Another data set for the experiment described in Fig 2A. (C) The minimum processing time of H_2_O_2_ treatment required for γ-H2AX signals to appear was 10 minutes. HeLa parental cells were treated with 500 μM H_2_O_2_ for indicated periods before fixation. Broken lines show nuclei. Scale bars, 20 μm.
(PDF)

S5 Fig Classification of tail lengths in comet assays. (A, B) The same data set shown in Fig 3A or 3B was divided into two classes. Fractions of each class per condition are represented by 100% stacked bar charts. Values in boxes represent the percentage fraction for each class. In each condition, numbers of analyzed comets (*n*) are shown above each bar. (A) Classification of the neutral comet assay data. Class 1, tail length shorter than 80 μm; Class 2, tail length longer than 80 μm. Chi-square test (with one degree of freedom) rejected the null hypothesis that the class distributions are the same between KD control and sh*STN1* cells (*p* = 2.4E-9). (B) Classification of the alkaline comet assay data. Class 1, tail length shorter than 155 μm; Class 2, tail length longer than 155 μm. Chi-square test (with one degree of freedom) rejected the null hypothesis that the class distributions are the same between KD control and sh*STN1* cells (*p* < 2.2E-16). (C, D) Histograms of comet tail lengths of H_2_O_2_-treated HeLa cells. The horizontal axis shows comet tail length (μm) and the vertical axis indicates counts for each binned value. Blue-colored regions indicate “Class 2” fractions.
(PDF)

S6 Fig Validation of mimosine-induced cell cycle arrest and synchronized release into S phase. (A) Experimental timeline. HeLa cells were treated with 500 μM mimosine for 23 hours to arrest them at the G1/S boundary [55]. At *t* = 0 hr, the arrested cells were synchronously released into S phase by replacing the medium with drug-free fresh medium. To monitor fractions of S-phase cells in the released cell population, pulse-labeling by EdU (incubation with 10 μM EdU for 10 min before fixation) was carried out at *t* = 0, 1, 2 and 4 hr, followed by conjugation with Alexa Flour 488 by Click chemistry (Click-iT EdU Cell Proliferation Kit for Imaging). EdU incorporation into nuclei was assessed by fluorescence microscopy. (B) Jitter plots showing the signal intensity of EdU (i.e., fluorescence intensity of Alexa Fluor 488 in a.u. [arbitrary units]). For each condition, the signal intensities of 500 randomly selected nuclei are shown. A nucleus with fluorescence intensity > 3,500 a.u. was defined to be EdU-positive. The EdU-positive rate per condition is shown above each lane. EdU (-), asynchronous cells without EdU labeling; EdU (+), asynchronous cells with EdU labeling. At *t* = 0 hr, all the analyzed nuclei were EdU-negative, demonstrating highly efficient cell cycle arrest at G1/S. As time elapsed, the fraction of EdU-positive nuclei gradually increased, indicating cell cycle progression into S phase with a modest synchronization rate. (C) Flow cytometric analysis of DNA content (PI fluorescence) in asynchronous (white) and mimosine-treated (colored) samples. After the release, cells were collected at *t* = 0 (red), 1 (green), 2 (blue), and 4 (purple) hours, respectively. The horizontal axis shows PI intensity (DNA content), and the vertical axis indicates cell counts for each intensity value.
(PDF)

S7 Fig DNA synthesis was impaired by H_2_O_2_ treatment. Jitter plots showing the signal intensity of EdU (i.e., fluorescence intensity of the conjugated Alexa Fluor 488 in a.u. [arbitrary units]). In each condition, the signal intensities of 700 randomly selected nuclei are shown. A nucleus with fluorescence intensity > 500 a.u. was defined to be EdU-positive. The EdU-positive rate per condition is shown above each lane. EdU (-), asynchronous cells without EdU labeling.
(PDF)

S8 Fig *STN1* knockdown decreased the number of RAD51 foci in H_2_O_2_-treated nuclei. The compiled data shown in Fig 6C are decomposed into the original two independent experiments, (A) and (B).
(PDF)

S9 Fig STN1 is required for nuclear RAD51 foci formation after oxidative damage in U2OS cells. The same set of experiments as in Fig 6 were performed using U2OS cells instead of HeLa cells. (A) Constitutive *STN1* knockdown in U2OS cells was evaluated by qRT-PCR. *STN1* RNA levels were normalized to *GAPDH* RNA levels. X-axis, cell lines; Y-axis, relative level of *STN1* RNA normalized to the KD control. The mean ± SEM is shown (n = 3). (B) Representative images of RAD51 foci in EdU-positive nuclei of U2OS cells. The enlarged area shown in the rightmost column is indicated by white squares. Scale bar: 10 μm. (C) Box-and-whisker plots showing the number of nuclear RAD51 foci per nucleus (Y-axis). At least 40 nuclei were randomly selected in each experimental condition. Numbers of analyzed nuclei (*n*) are shown at the top of each lane. *p*-values for Mann–Whitney *U* tests are shown above the indicated lanes.
(PDF)

S10 Fig Validation of RAD51 inhibition by B02. (A) The experimental timeline. HeLa cells were treated with RAD51 inhibitor B02 starting one hour before the beginning of Etposide treatment (25 μM, 1 hour). (B) Representative images of RAD51 foci and γ-H2AX signals in HeLa cell nuclei. The enlarged area shown in the rightmost column is indicated by white squares in the RAD51 column. RAD51 foci formation induced by etoposide treatment was suppressed by B02 treatment. Scale bars, 10 μm. (C) Distributions of the number of RAD51 foci per nucleus in HeLa cells. *p*-values for Mann–Whitney *U* tests are shown above the indicated lanes. (D) Distributions of the number of RAD51 foci per nucleus in U2OS cells. *p*-values for Mann–Whitney *U* tests are shown above the indicated lanes.
(PDF)

S11 Fig Graphical summary. Oxidative stress by hydrogen peroxide can cause a variety of DNA damages including single-strand breaks, double-strand breaks, and base modifications. In S phase, such DNA lesions can cause replication fork stalling and/or generate one-ended DSBs through fork collisions with SSB sites. RAD51 is crucial in both restarting stalled forks and initiating HR-dependent repair of the collapsed forks. The CST complex could contribute to efficient RAD51 loading onto the damaged sites by protecting and stabilizing the exposed ssDNA (which is a substrate for RAD51 filament formation) and/or by directly recruiting RAD51. See Discussion for details.
(PDF)

S1 Table List of antibodies used in this study. (XLSX)
