# Supplementary figures and images for: The CST complex facilitates cell survival under oxidative genotoxic stress
Source: PLoS One. 2023 Aug 17;18(8):e0289304. doi: 10.1371/journal.pone.0289304 (PMC10434909; doi:10.1371/journal.pone.0289304)

**A**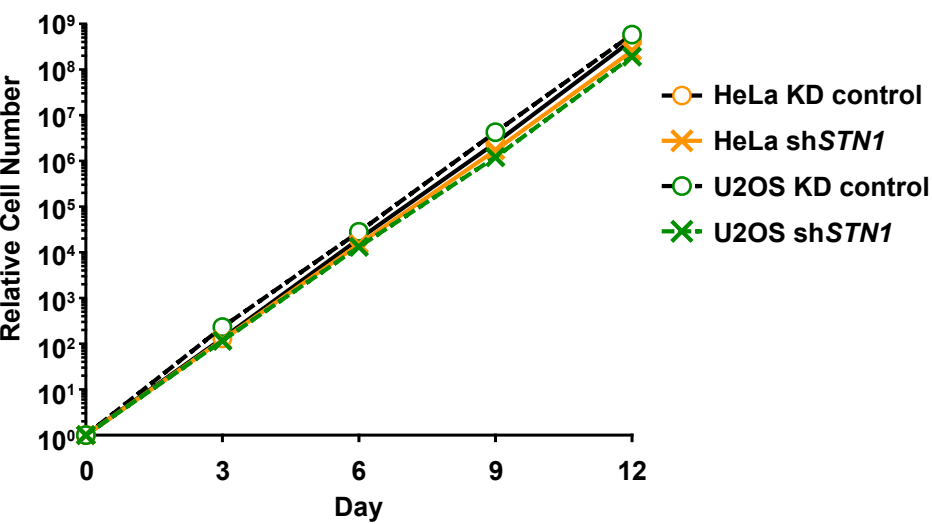**B**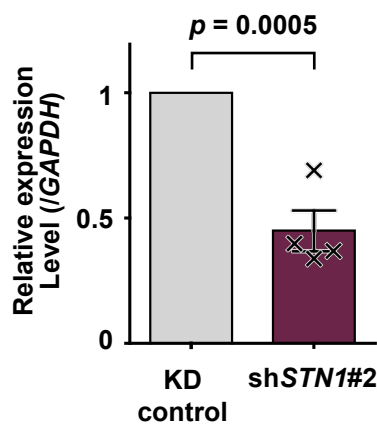**C**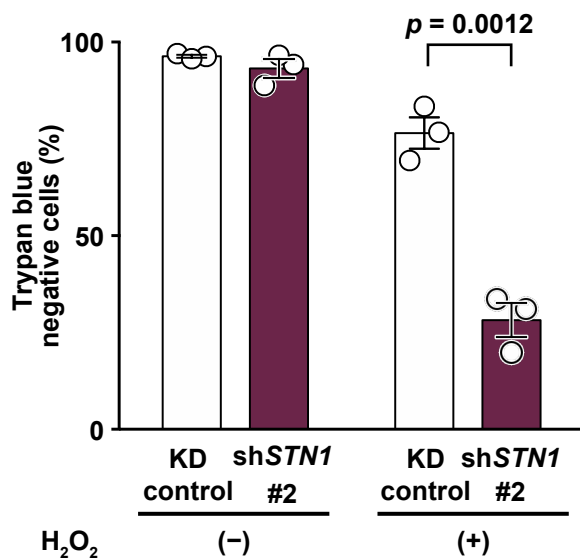**D**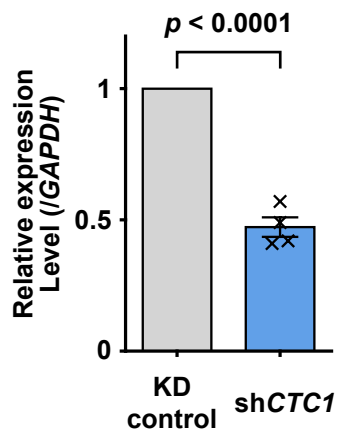**E**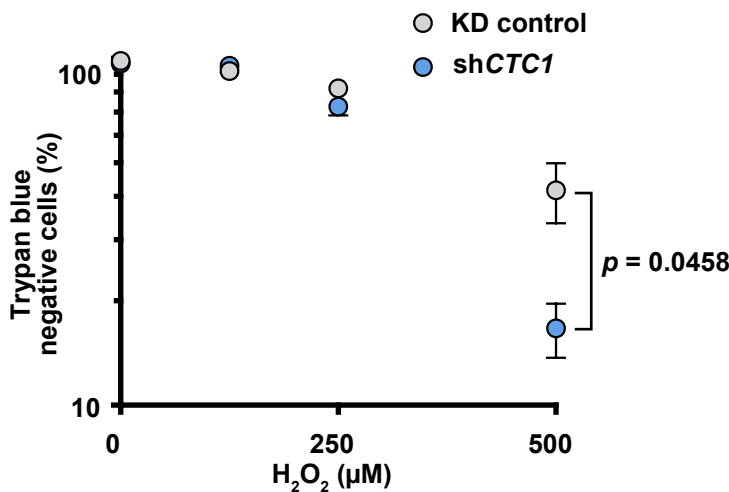

Supplement: S1 Fig — (A) Growth curve of HeLa or U2OS shSTN1 cells. (B) Constitutive STN1 knockdown in HeLa cells using the shSTN1#2 construct was evaluated by qRT-PCR. STN1 RNA levels were normalized to GAPDH RNA levels. X-axis, cell lines. Y-axis, relative level of STN1 RNA normalized to the KD control. Error-bars represent SEM. (C) Viability assay of HeLa cells treated with 500 μM of H2O2 for 2 hours. Viability was measured 2 days after the H2O2 treatment by trypan blue staining. Data from three biological replicates are shown with mean values (boxes). The p-value for the unpaired t-test is shown above the indicated bars. (D) Constitutive CTC1 knockdown in HeLa cells was evaluated by qRT-PCR. CTC1 RNA levels were normalized to GAPDH RNA levels. X-axis, cell lines. Y-axis, relative level of CTC1 RNA normalized to the KD control. Mean ± SEM (n = 4). (E) Viability assay of HeLa cells treated with the indicated doses of H2O2 for 2 hours. Error-bars represent SEM. The p-value for the unpaired t-test is shown. We note that it was previously reported that lack of any CST component results in unstable binding between CST components and ssDNA [3, 54]. Thus, in this research, we preferentially used cultured cells depleted of STN1, which bridges CTC1 and TEN1. (PDF) [file pone.0289304.s001.pdf]

**A**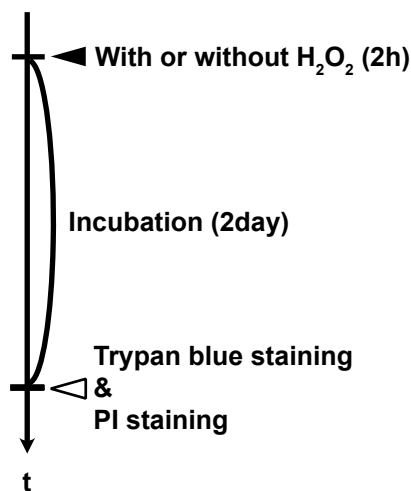**C**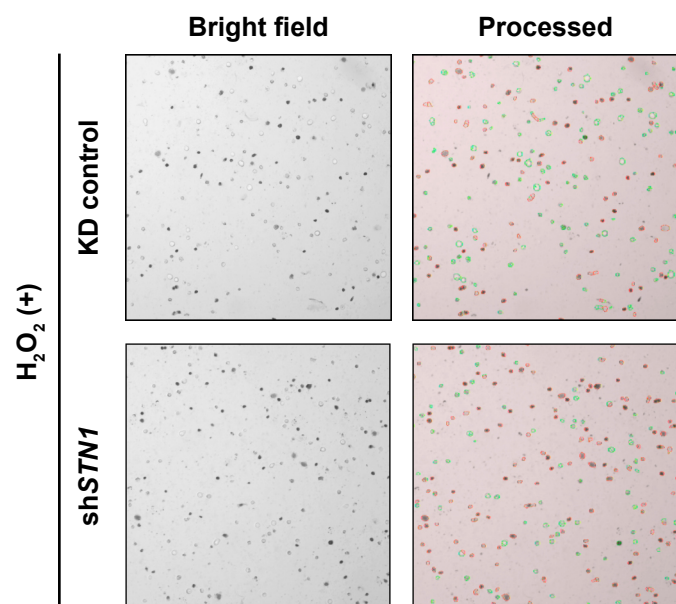**B**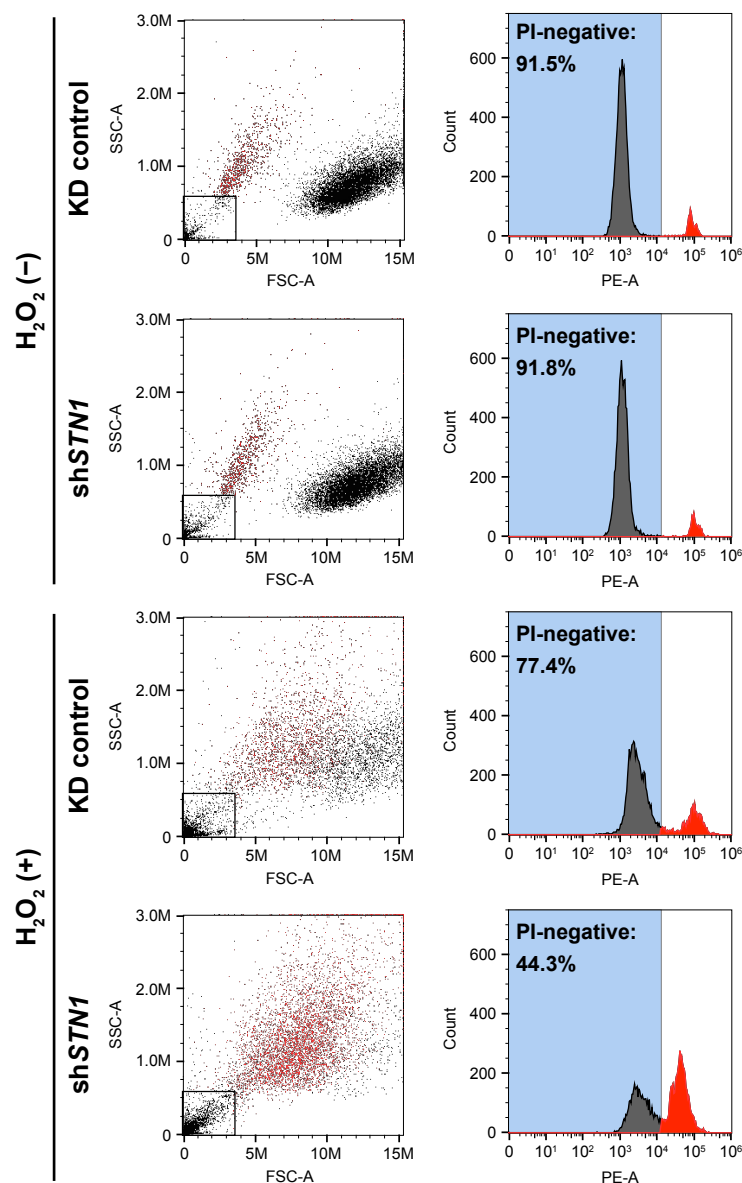**D**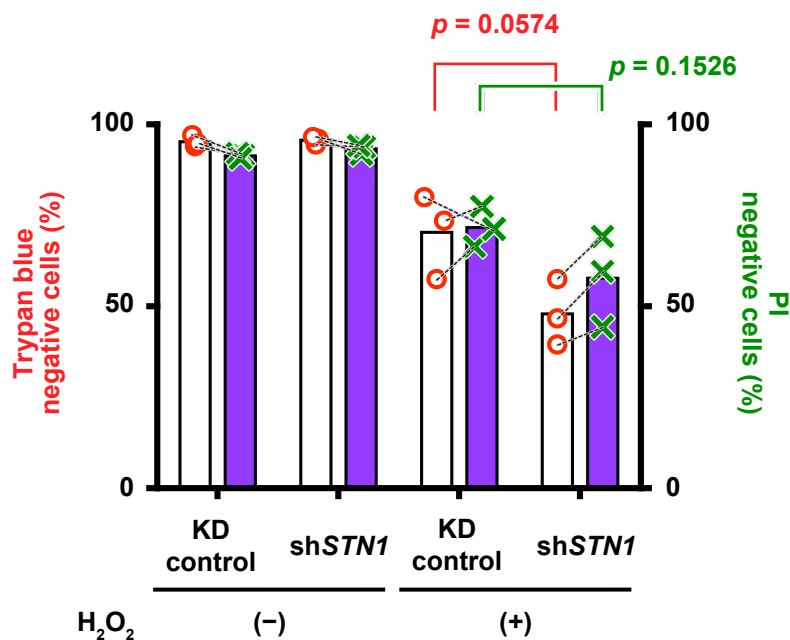

Supplement: S2 Fig — (A) Experimental design. HeLa cells were treated with H2O2 (500 μM, 2 hours). Immediately after washing out the H2O2, the cells were incubated for two days in standard medium. Finally, all cells were collected, and half of the sample was subjected to Trypan blue staining, while the other half was subjected to PI staining. (B) Representative data set of flow cytometric analysis of PI-stained cells. Left-side panels are representative forward scatter versus side scatter dot plots, showing 10,000 cells in each panel. The square gates at the bottom-left corners show debris that was excluded from the analysis. The red dots are PI-positive cells, which were determined according to the histograms shown in the right side. In the histograms, blue-colored regions indicate PI-negative fractions. (C) Representative images of trypan-blue-stained cells taken by a Countess automated cell counter. Bright-field images were automatically processed by the equipment software to identify stained (dead) and unstained (alive) cells. In the processed images, dead and live cells are indicated by red and green, respectively. (D) Comparison between trypan blue staining and PI staining. In each experimental condition, data points for the estimated viabilities with the two methods are connected by broken lines. Data from three biological replicates are shown with mean values (boxes). p-values for unpaired t-tests are shown above the respective paired bars. (PDF) [file pone.0289304.s002.pdf]

**A**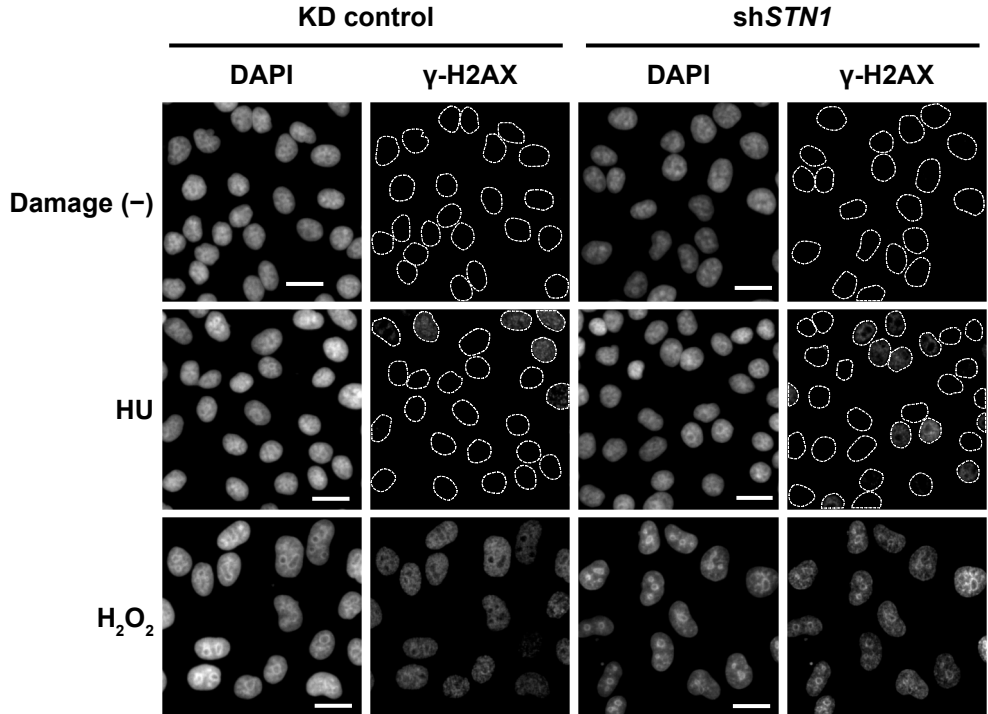**B**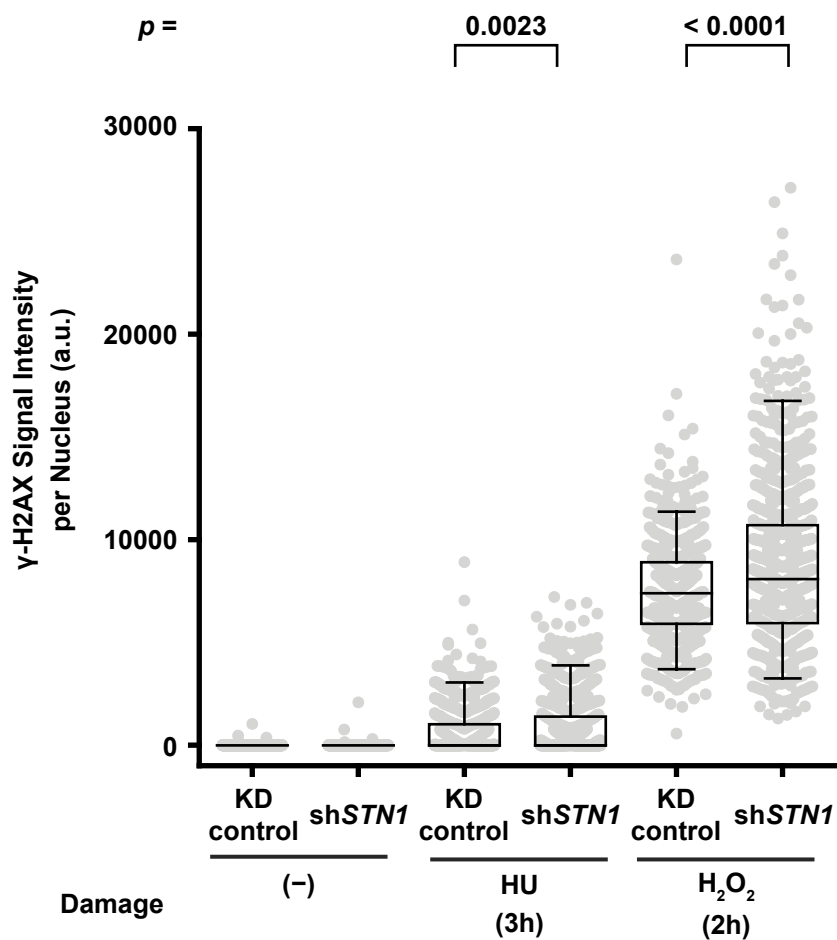

Supplement: S3 Fig — (A) Representative images of an indirect immunofluorescence assay for γ-H2AX in untreated, HU-, or H2O2-treated HeLa cells. Broken lines show nuclei of untreated or HU-treated cells. Scale bars, 20 μm. (B) Box-and-whisker plots showing the signal intensity of γ-H2AX (i.e. fluorescence intensity of Alexa Fluor 488 in a.u. [arbitrary units]) in the nuclei. In each condition, the signal intensities of 800 randomly selected nuclei are shown. p-values for unpaired t-tests are shown above the respective bars. (PDF) [file pone.0289304.s003.pdf]

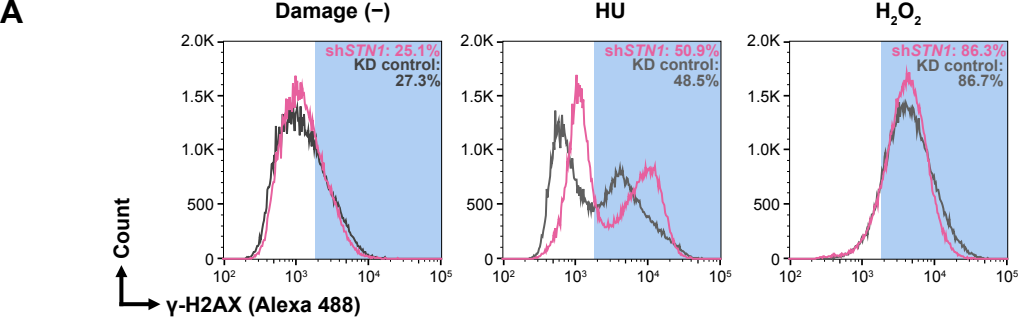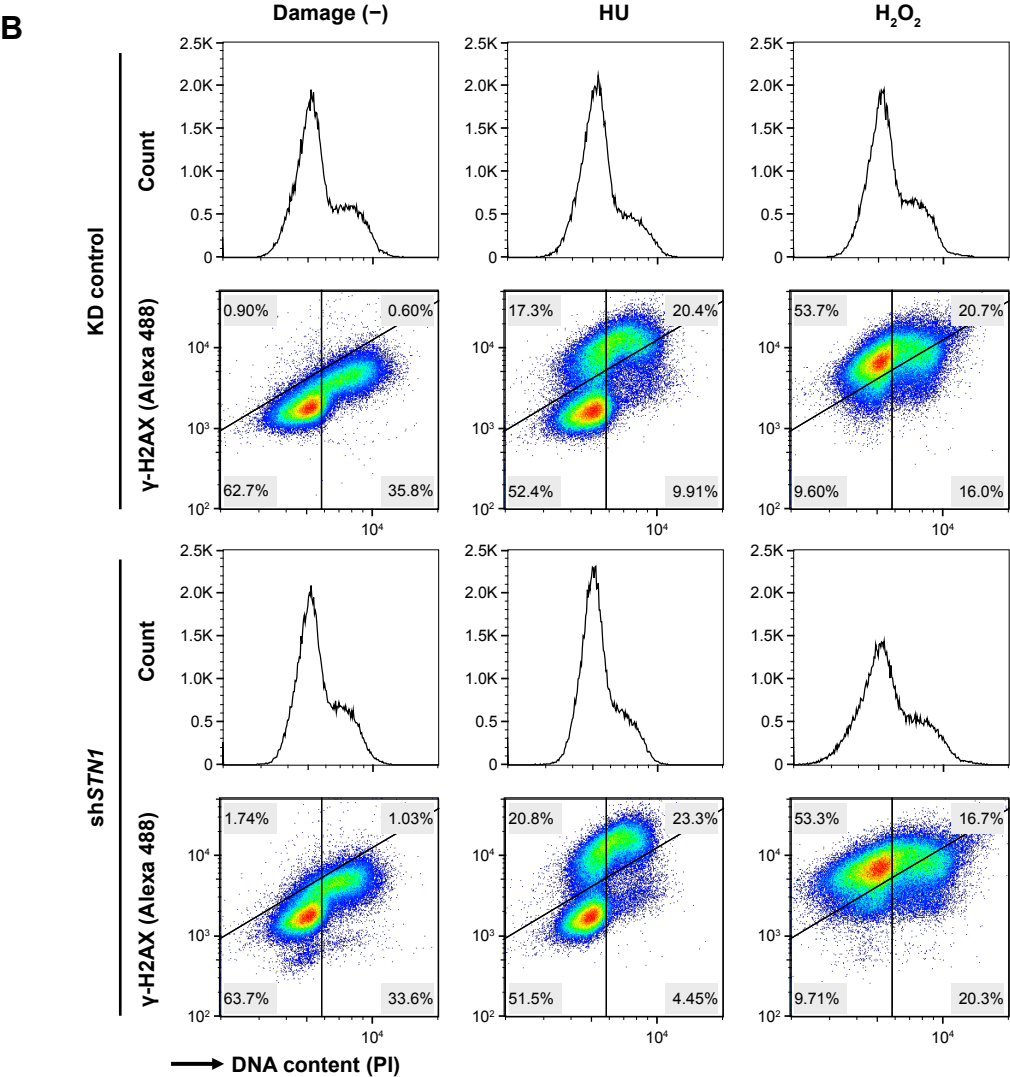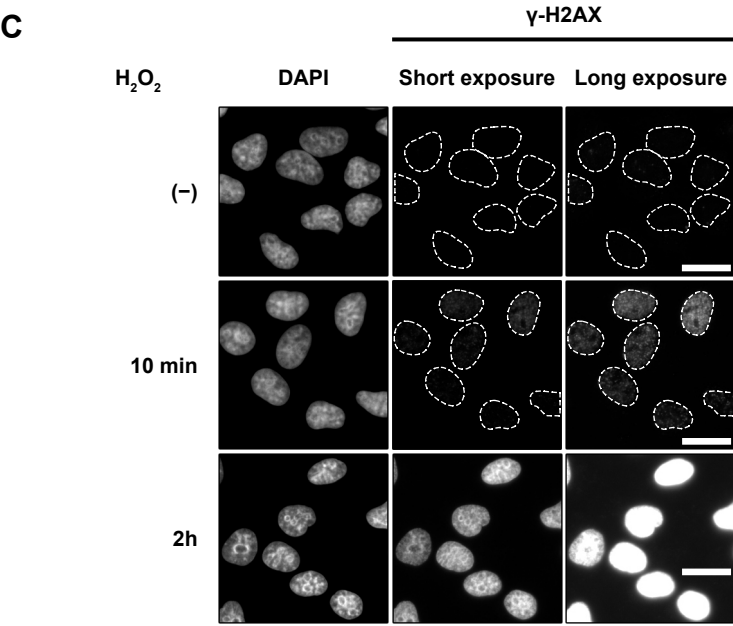

Supplement: S4 Fig — (A) Histograms of Alexa488 intensity in undamaged, HU- or H2O2-treated HeLa cells. The horizontal axis shows Alexa488 fluorescence (γ-H2AX level), and the vertical axis indicates cell counts for each intensity value. Blue-colored regions indicate γ-H2AX positive fractions. (B) Another data set for the experiment described in Fig 2A. (C) The minimum processing time of H2O2 treatment required for γ-H2AX signals to appear was 10 minutes. HeLa parental cells were treated with 500 μM H2O2 for indicated periods before fixation. Broken lines show nuclei. Scale bars, 20 μm. (PDF) [file pone.0289304.s004.pdf]

**A**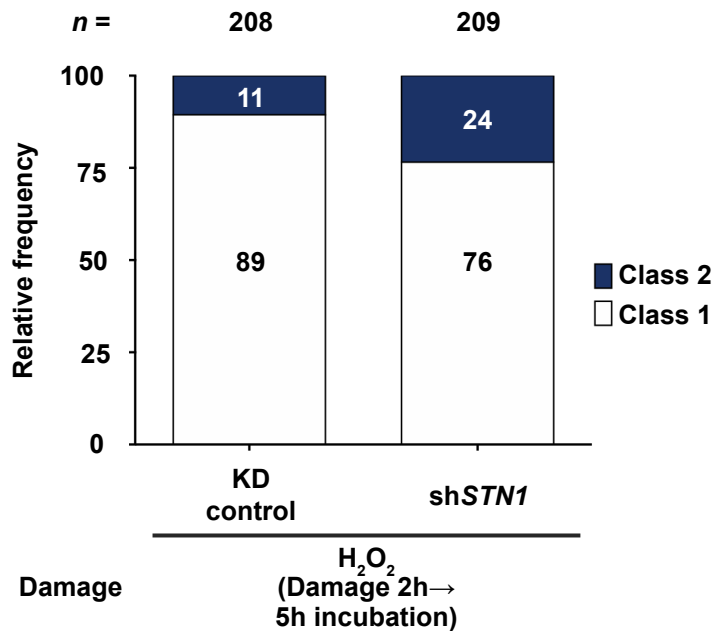**B**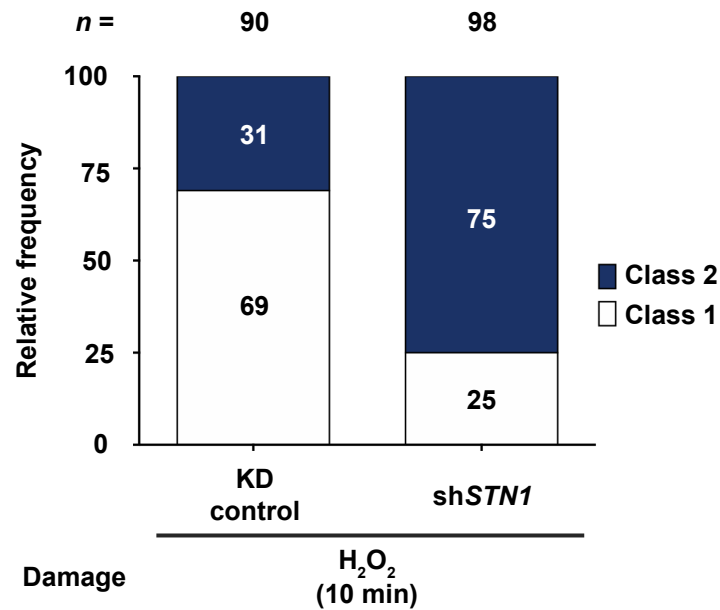**C**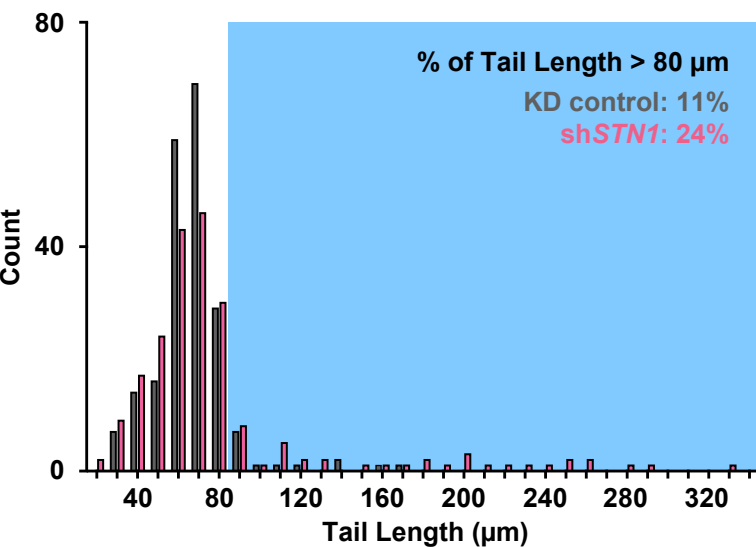**D**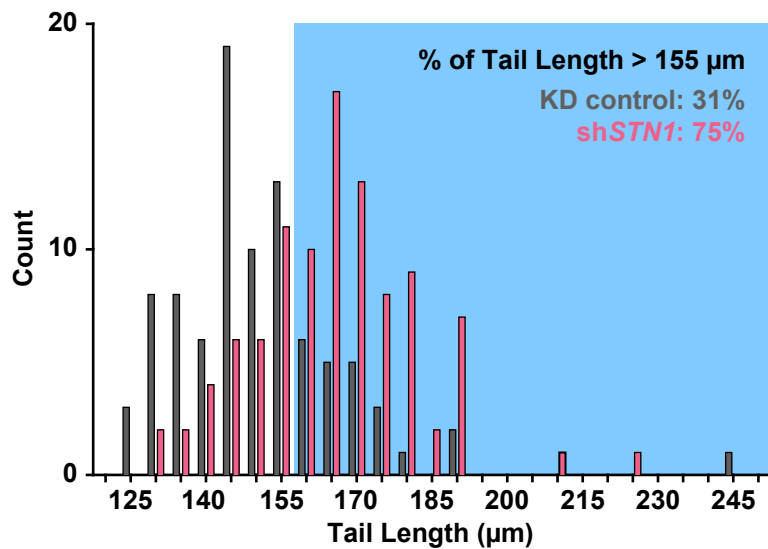

Supplement: S5 Fig — (A, B) The same data set shown in Fig 3A or 3B was divided into two classes. Fractions of each class per condition are represented by 100% stacked bar charts. Values in boxes represent the percentage fraction for each class. In each condition, numbers of analyzed comets (n) are shown above each bar. (A) Classification of the neutral comet assay data. Class 1, tail length shorter than 80 μm; Class 2, tail length longer than 80 μm. Chi-square test (with one degree of freedom) rejected the null hypothesis that the class distributions are the same between KD control and shSTN1 cells (p = 2.4E-9). (B) Classification of the alkaline comet assay data. Class 1, tail length shorter than 155 μm; Class 2, tail length longer than 155 μm. Chi-square test (with one degree of freedom) rejected the null hypothesis that the class distributions are the same between KD control and shSTN1 cells (p < 2.2E-16). (C, D) Histograms of comet tail lengths of H2O2-treated HeLa cells. The horizontal axis shows comet tail length (μm) and the vertical axis indicates counts for each binned value. Blue-colored regions indicate “Class 2” fractions. (PDF) [file pone.0289304.s005.pdf]

A

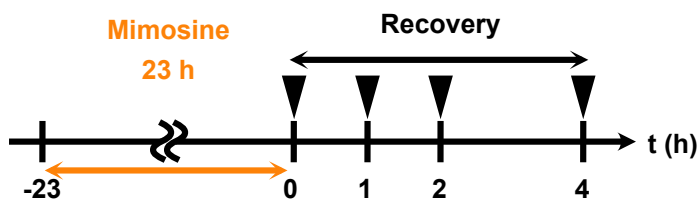

▼ : EdU (10-min pulse-label & fixation), FACS (harvest cells & fixation)

B

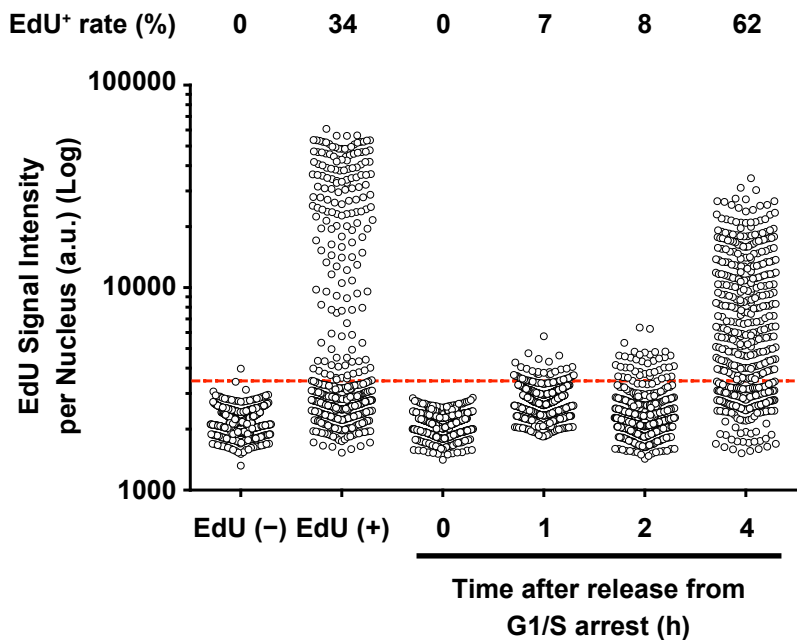

C

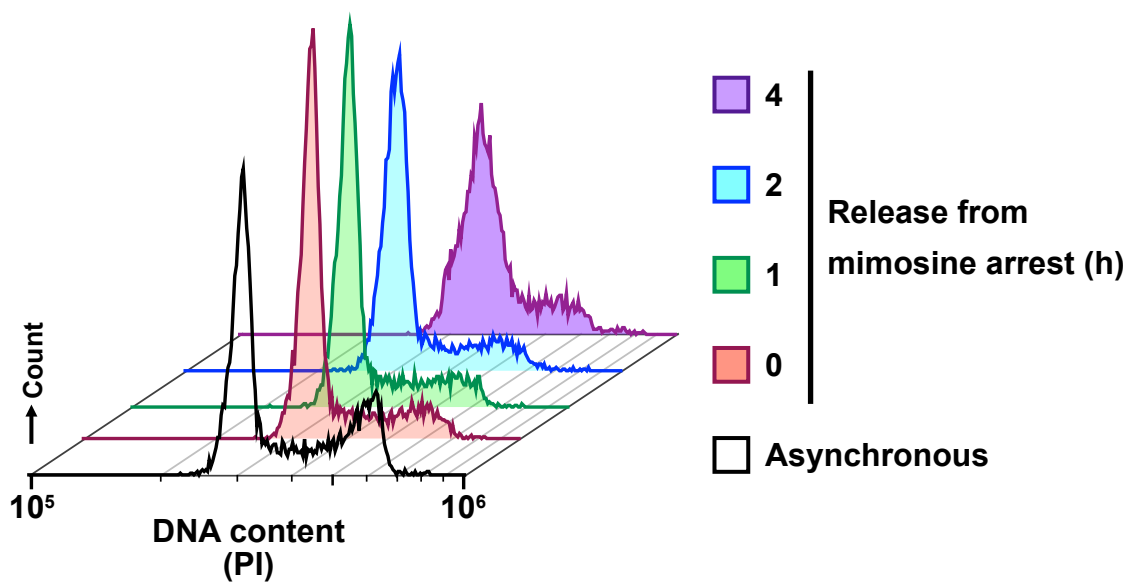

Supplement: S6 Fig — (A) Experimental timeline. HeLa cells were treated with 500 μM mimosine for 23 hours to arrest them at the G1/S boundary [55]. At t = 0 hr, the arrested cells were synchronously released into S phase by replacing the medium with drug-free fresh medium. To monitor fractions of S-phase cells in the released cell population, pulse-labeling by EdU (incubation with 10 μM EdU for 10 min before fixation) was carried out at t = 0, 1, 2 and 4 hr, followed by conjugation with Alexa Flour 488 by Click chemistry (Click-iT EdU Cell Proliferation Kit for Imaging). EdU incorporation into nuclei was assessed by fluorescence microscopy. (B) Jitter plots showing the signal intensity of EdU (i.e., fluorescence intensity of Alexa Fluor 488 in a.u. [arbitrary units]). For each condition, the signal intensities of 500 randomly selected nuclei are shown. A nucleus with fluorescence intensity > 3,500 a.u. was defined to be EdU-positive. The EdU-positive rate per condition is shown above each lane. EdU (-), asynchronous cells without EdU labeling; EdU (+), asynchronous cells with EdU labeling. At t = 0 hr, all the analyzed nuclei were EdU-negative, demonstrating highly efficient cell cycle arrest at G1/S. As time elapsed, the fraction of EdU-positive nuclei gradually increased, indicating cell cycle progression into S phase with a modest synchronization rate. (C) Flow cytometric analysis of DNA content (PI fluorescence) in asynchronous (white) and mimosine-treated (colored) samples. After the release, cells were collected at t = 0 (red), 1 (green), 2 (blue), and 4 (purple) hours, respectively. The horizontal axis shows PI intensity (DNA content), and the vertical axis indicates cell counts for each intensity value. (PDF) [file pone.0289304.s006.pdf]

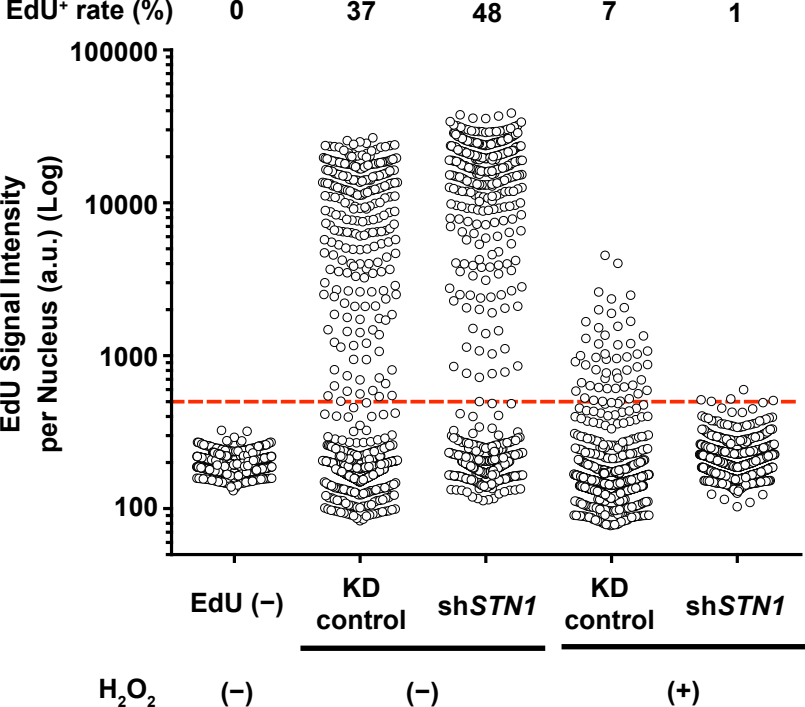

Supplement: S7 Fig — Jitter plots showing the signal intensity of EdU (i.e., fluorescence intensity of the conjugated Alexa Fluor 488 in a.u. [arbitrary units]). In each condition, the signal intensities of 700 randomly selected nuclei are shown. A nucleus with fluorescence intensity > 500 a.u. was defined to be EdU-positive. The EdU-positive rate per condition is shown above each lane. EdU (-), asynchronous cells without EdU labeling. (PDF) [file pone.0289304.s007.pdf]

**A**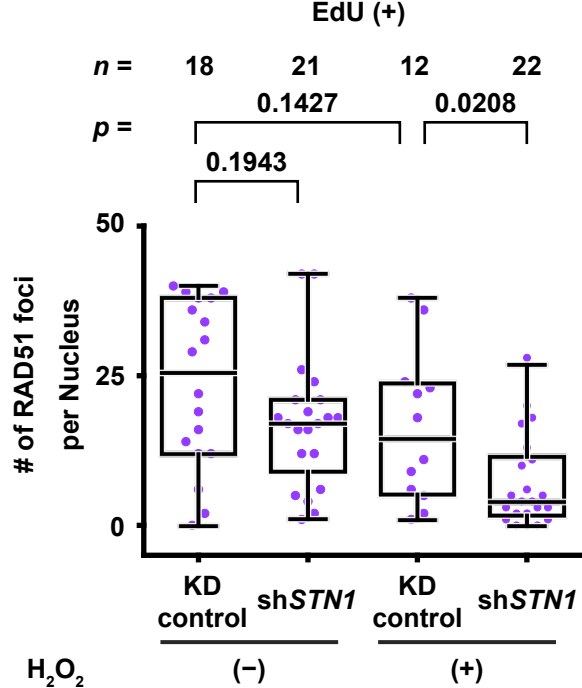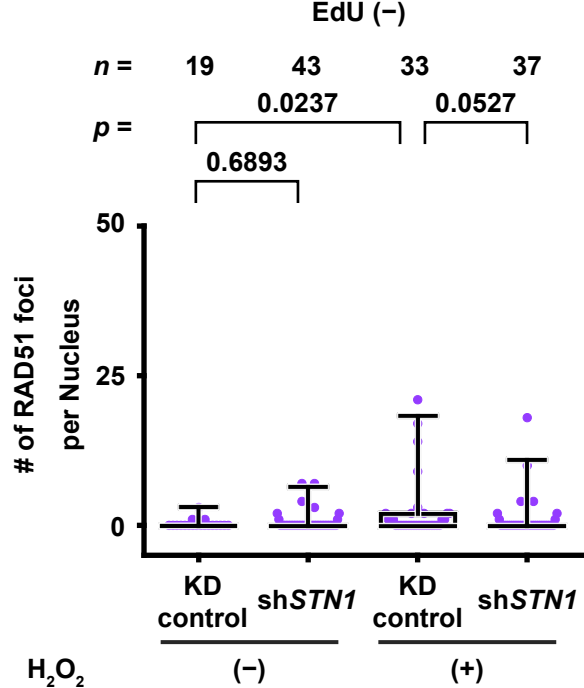**B**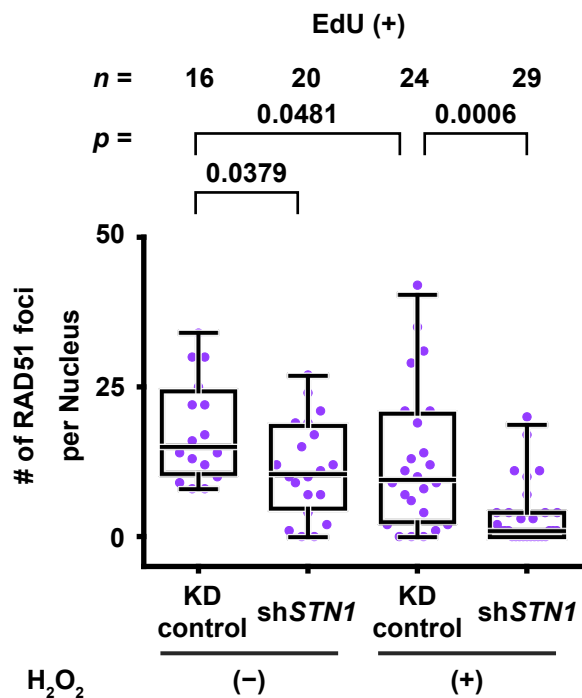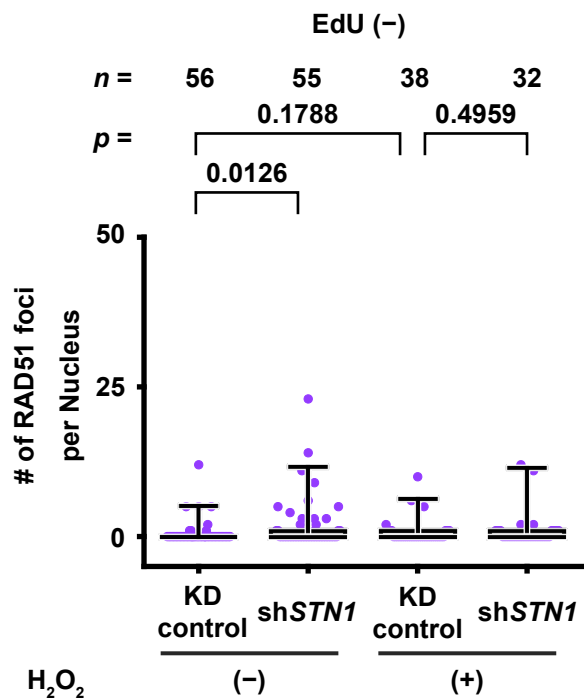

Supplement: S8 Fig — The compiled data shown in Fig 6C are decomposed into the original two independent experiments, (A) and (B). (PDF) [file pone.0289304.s008.pdf]

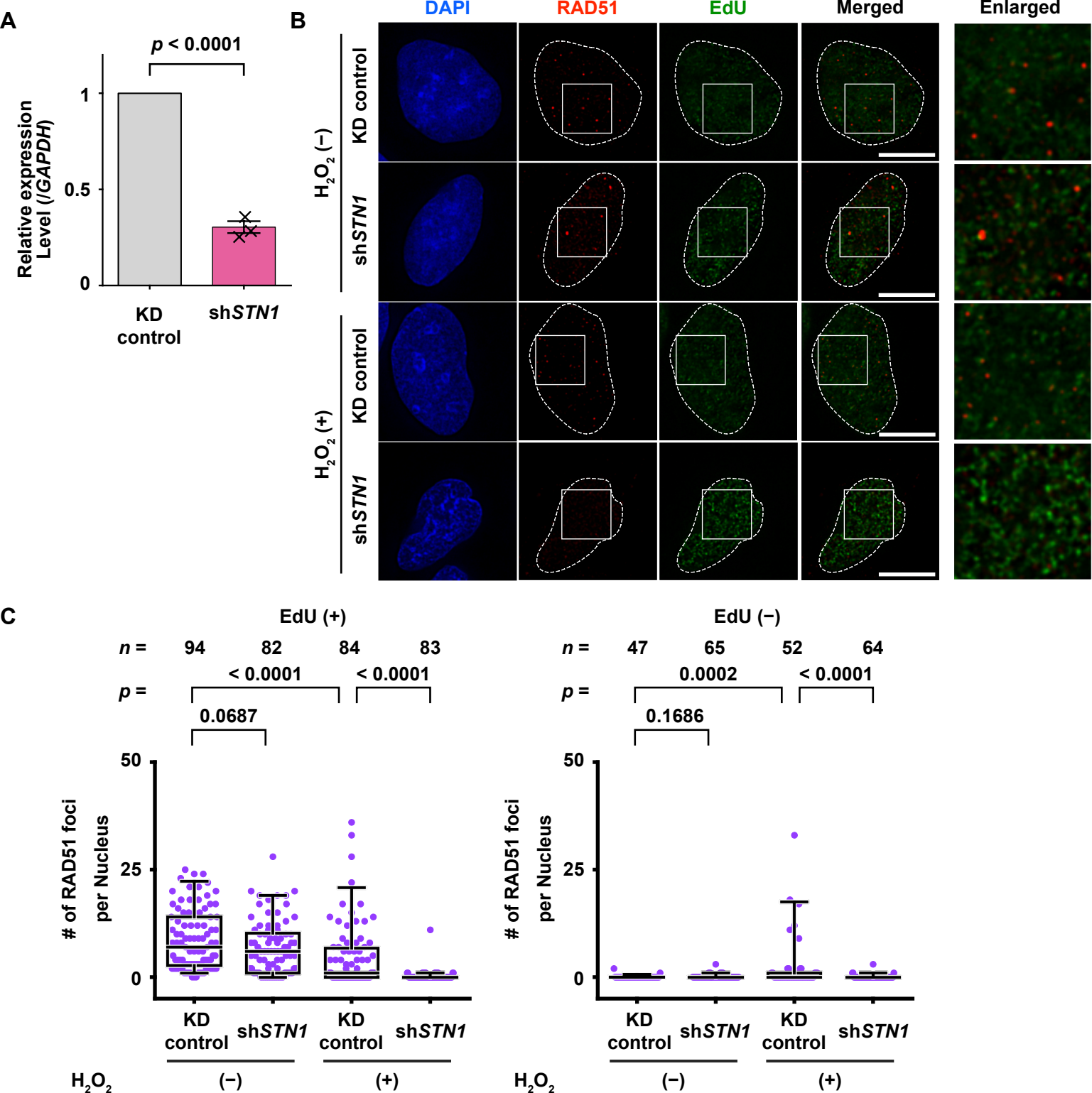

Supplement: S9 Fig — The same set of experiments as in Fig 6 were performed using U2OS cells instead of HeLa cells. (A) Constitutive STN1 knockdown in U2OS cells was evaluated by qRT-PCR. STN1 RNA levels were normalized to GAPDH RNA levels. X-axis, cell lines; Y-axis, relative level of STN1 RNA normalized to the KD control. The mean ± SEM is shown (n = 3). (B) Representative images of RAD51 foci in EdU-positive nuclei of U2OS cells. The enlarged area shown in the rightmost column is indicated by white squares. Scale bar: 10 μm. (C) Box-and-whisker plots showing the number of nuclear RAD51 foci per nucleus (Y-axis). At least 40 nuclei were randomly selected in each experimental condition. Numbers of analyzed nuclei (n) are shown at the top of each lane. p-values for Mann–Whitney U tests are shown above the indicated lanes. (PDF) [file pone.0289304.s009.pdf]

**A**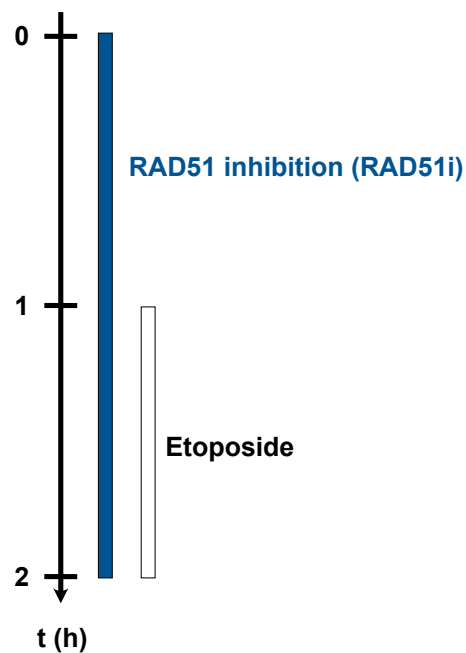**B**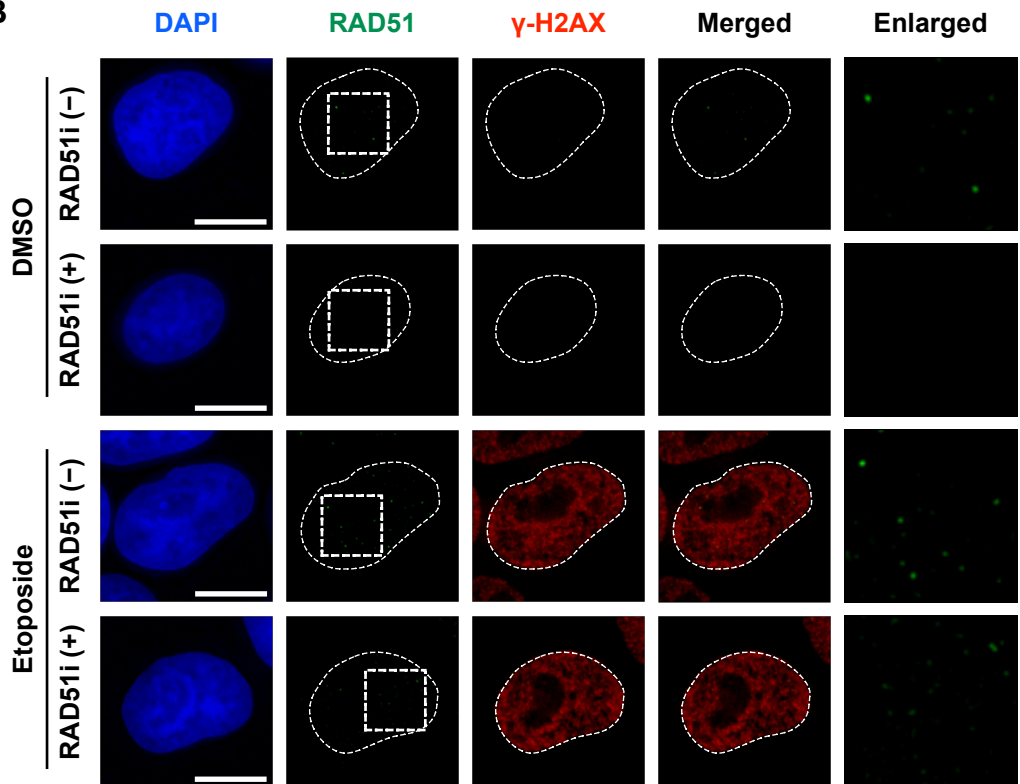**C**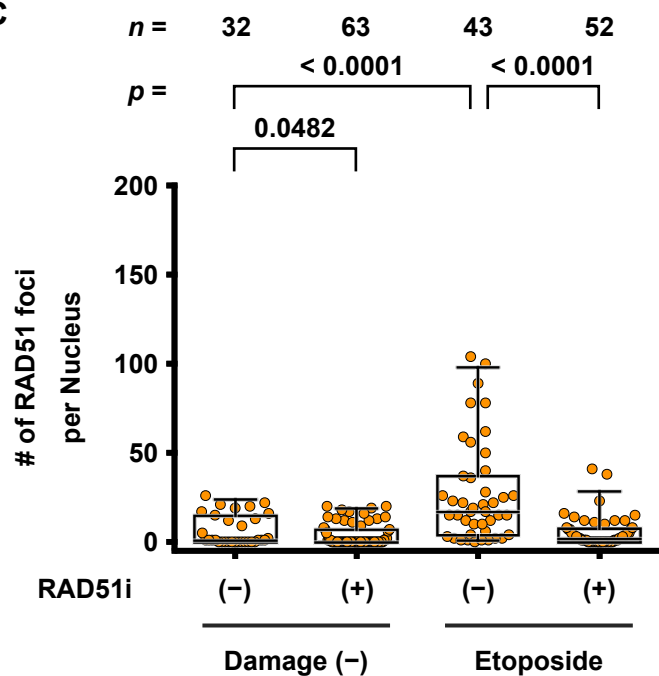**D**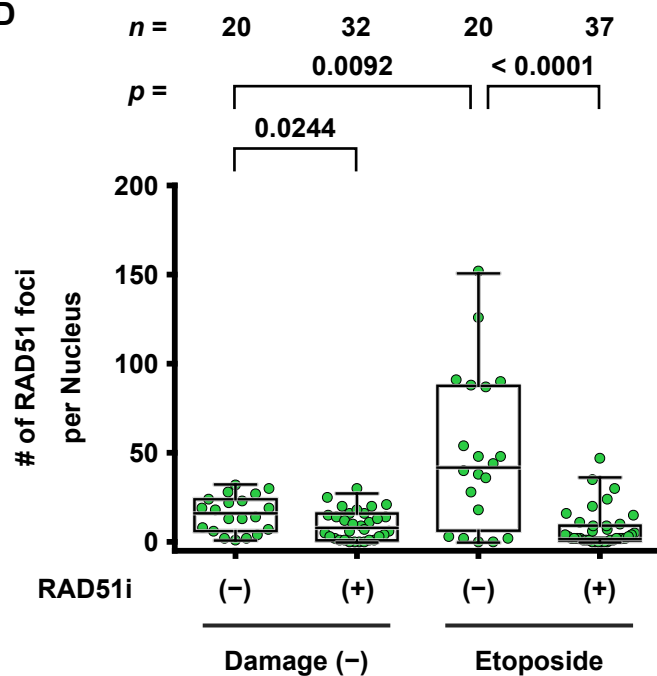

Supplement: S10 Fig — (A) The experimental timeline. HeLa cells were treated with RAD51 inhibitor B02 starting one hour before the beginning of Etposide treatment (25 μM, 1 hour). (B) Representative images of RAD51 foci and γ-H2AX signals in HeLa cell nuclei. The enlarged area shown in the rightmost column is indicated by white squares in the RAD51 column. RAD51 foci formation induced by etoposide treatment was suppressed by B02 treatment. Scale bars, 10 μm. (C) Distributions of the number of RAD51 foci per nucleus in HeLa cells. p-values for Mann–Whitney U tests are shown above the indicated lanes. (D) Distributions of the number of RAD51 foci per nucleus in U2OS cells. p-values for Mann–Whitney U tests are shown above the indicated lanes. (PDF) [file pone.0289304.s010.pdf]
