# Supplementary material for: The CST complex facilitates cell survival under oxidative genotoxic stress
Source: PLoS One. 2023 Aug 17;18(8):e0289304. doi: 10.1371/journal.pone.0289304 (PMC10434909; doi:10.1371/journal.pone.0289304)
Supplement: S11 Fig — Oxidative stress by hydrogen peroxide can cause a variety of DNA damages including single-strand breaks, double-strand breaks, and base modifications. In S phase, such DNA lesions can cause replication fork stalling and/or generate one-ended DSBs through fork collisions with SSB sites. RAD51 is crucial in both restarting stalled forks and initiating HR-dependent repair of the collapsed forks. The CST complex could contribute to efficient RAD51 loading onto the damaged sites by protecting and stabilizing the exposed ssDNA (which is a substrate for RAD51 filament formation) and/or by directly recruiting RAD51. See Discussion for details. (PDF) [file pone.0289304.s011.pdf]

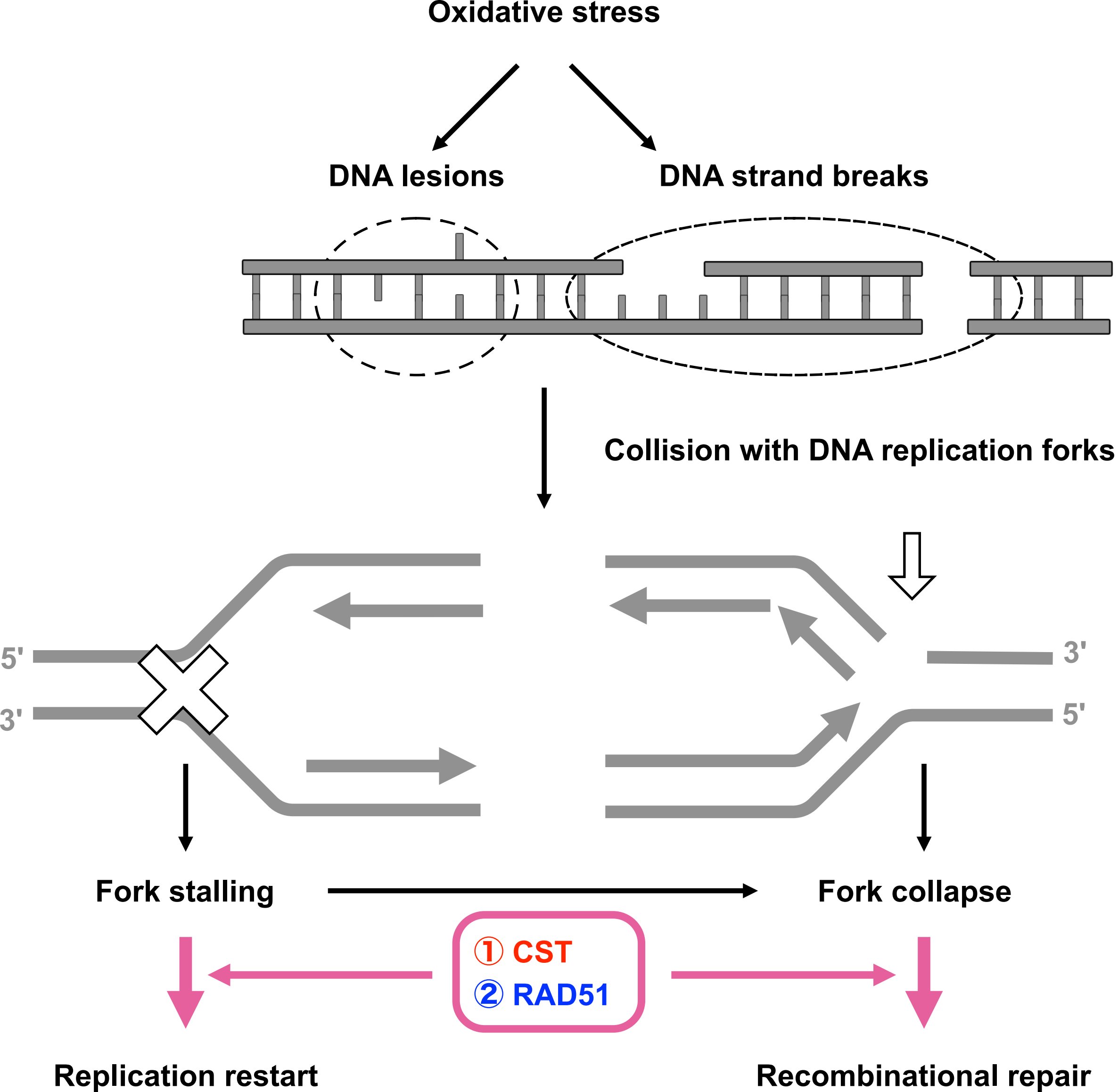

Oxidative stress
DNA lesions
DNA strand breaks
Collision with DNA replication forks
5'
3'
3'
5'
Fork stalling
Fork collapse
① CST
② RAD51
Replication restart
Recombinational repair
